# High Affinity Iron Acquisition Systems Facilitate but Are Not Essential for Colonization of Chickens by *Salmonella* Enteritidis

**DOI:** 10.3389/fmicb.2022.824052

**Published:** 2022-03-03

**Authors:** Dinesh H. Wellawa, Po-King S. Lam, Aaron P. White, Susantha Gomis, Brenda Allan, Wolfgang Köster

**Affiliations:** ^1^Vaccine and Infectious Disease Organization, University of Saskatchewan, Saskatoon, SK, Canada; ^2^Department of Veterinary Microbiology, Western College of Veterinary Medicine, University of Saskatchewan, Saskatoon, SK, Canada; ^3^Department of Veterinary Pathology, Western College of Veterinary Medicine, University of Saskatchewan, Saskatoon, SK, Canada

**Keywords:** enterobactin, *Salmonella*, FeoABC transporter, chicken, iron uptake

## Abstract

The roles of TonB mediated Fe^3+^ (ferric iron) uptake via enterobactin (involving biosynthesis genes *entABCDEF*) and Fe^2+^ (ferrous iron) uptake through the FeoABC transporter are poorly defined in the context of chicken-*Salmonella* interactions. Both uptake systems are believed to be the major contributors of iron supply in the *Salmonella* life cycle. Current evidence suggests that these iron uptake systems play a major role in pathogenesis in mammals and as such, they represent promising antibacterial targets with therapeutic potential. We investigated the role of these iron uptake mechanisms regarding the ability of *Salmonella* Enteritidis (SEn) strains to colonize in a chicken infection model. Further we constructed a bioluminescent reporter to sense iron limitation during gastrointestinal colonization of *Salmonella* in chicken via *ex vivo* imaging. Our data indicated that there is some redundancy between the ferric and ferrous iron uptake mechanisms regarding iron acquisition during SEn pathogenesis in chicken. We believe that this redundancy of iron acquisition in the host reservoir may be the consequence of adaptation to unique avian environments, and thus warrants further investigation. To our knowledge, this the first report providing direct evidence that both enterobactin synthesis and FeoABC mediated iron uptake contribute to the virulence of SEn in chickens.

## Introduction

Iron is considered as an indispensable element during the life cycle of *Salmonella*. It is critical during aerobic respiration and acts as a cofactor for numerous enzymatic processes in the bacterial cell including DNA and RNA synthesis (Andrews et al., 2003). Iron can exist in a wide range of oxidation states with +2 and +3 as the most common. In anaerobic and/low-pH environments, Fe^2+^ is the stable form which is relatively soluble at physiological conditions (Andrews et al., 2003; Cartron et al., 2006). Compared to ferrous iron, Fe^3+^ predominates under aerobic conditions and is insoluble at physiological pH (>3.5). To adopt these interchangeable valences and chemical properties of iron, *Salmonella* encodes several iron acquisition systems.

**FIGURE 1:**
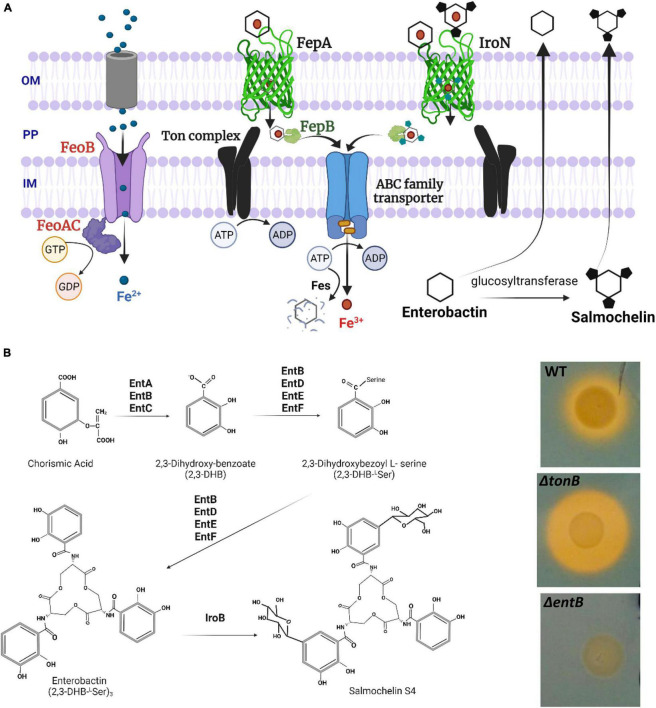
**(A)** Ferrous iron and catecholate siderophore chelated ferric iron uptake via high affinity acquisition systems in *Salmonella.* OM, outer membrane; PP, periplasm; IM, inner membrane. Ton complex components (TonB-ExbB-ExbD) transduce the proton motive force from the inner membrane to outer membrane receptors to translocate the ferric-siderophore into the PP. FepB is the periplasmic binding protein to shuttle siderophores via ABC family transporter (FecC, FecD, FecE) into the cytoplasm. Fes (ferric enterochelin esterase) hydrolyzes the catecholate siderophore to release Fe^3+^. Not shown components involved in the transport of hydroxamate type ferric-siderophores and iron-dicitrate. **(B)** Siderophore biosynthesis in key steps. Enzymes involved in enterobactin synthesis are encoded by *entCEBA* operon and *entF* transcribed separately. IroB represents a glucosyltransferase. Detection of siderophore production using chrome azurol S agar plate is shown on the right side. Five μL of overnight cultures were spotted onto the agar plate and incubated at 37°C overnight. A yellow halo is indicative of siderophore mediated ferric iron chelation. WT, wild type (LS101 SEn).

Fe^2+^ shows relatively high affinity to the FeoABC import channel located in the inner membrane of the bacterial cell (K_m_ ∼ 0.5 μM) (Cartron et al., 2006). Since Fe^2+^ is more water soluble, it reaches threshold concentrations for diffusion through outer membrane porins into the periplasm before getting channeled through the FeoABC system (Figure 1A). The unique nature of FeoABC is that it is dedicated to ferrous iron uptake without preference for any other metal (Hantke, 2003; Lau et al., 2016; Sestok et al., 2018). The transmembrane domain of FeoB provides a gated channel which allows ferrous iron to pass through once the membrane potential has changed (Figure 1A). This change in the membrane potential is driven by slow GTP hydrolyzation and fast GDP release at the N terminal part of FeoB (Hantke, 2003; Lau et al., 2016). Accessory proteins such as FeoA and FeoC are believed to interact with FeoB to modulate the GTP hydrolyzation process and the rate of ferrous iron uptake according to cellular demand (Hantke, 2003). Fe^2+^ can also be shuttled into the cytoplasm of *Salmonella* using other metal transporters such as MntH and SitABCD located in the inner membrane. In contrast to FeoABC, both MntH and SitABCD have high affinity (K_a_) to Mn^2+^ (∼0.1 μM) rather than to ferrous iron (∼3–10 μM) (Kehres et al., 2002). There is compelling evidence that ferrous iron uptake contributes to the virulence of *Salmonella* Typhimurium (STm) in the murine model (Tsolis et al., 1996; Janakiraman and Slauch, 2000; Boyer et al., 2002; Zaharik et al., 2004; Nagy et al., 2014; Cunrath and Bumann, 2019). However, requirement of FeoABC alone for the virulence of STm in pathogenesis in mice remains intriguing due to variation in strain types, genetic background of mice and inoculum routes used. For example, in co-infection experiments with the wild type, lack of FeoABC alone reduced the fecal shedding of STm strains 14028 (chicken isolate), SL1344 (calf isolate) in *Salmonella* susceptible (BALB/c) and resistant (Sv129S6-Nramp^+/+^) mice backgrounds respectively (Tsolis et al., 1996; Nagy et al., 2014). However, oral challenge with the Δ*feo* mutant of SL1344 strain alone, did not reduce fecal shedding or decrease systemic infections in resistant mice (Sv129S6-Nramp^+/+^) (Nagy et al., 2014). Further, given intravenously, STm Keller strain (“pigeon variant”) carrying Δ*feo* was virulent and caused mortality in susceptible mice (Sv129S6-Nramp^–/–^) (Boyer et al., 2002). In contrast all susceptible mice were able to survive an intravenous challenge of STm carrying deletions of Δ*feoΔsit* or Δ*feoΔmntH*, pointing to redundancy between ferrous iron acquisition systems in regard to virulence (Boyer et al., 2002).

Fe^3+^ is the major iron form in the environment, commonly found as ferric hydroxides. Ferric hydroxides are extremely insoluble at physiological pH and free Fe^3+^ is estimated to be <1 μM (Ehrlich et al., 2002). At this concentration, passive diffusion of ferric iron through outer membrane porins will be unlikely. Ferric iron is enriched in soil or marine environments but tightly bound to humic substances (consisting of phenols, aromatic amino acids, quinones, fatty acids etc.); hence bioavailability for *Salmonella* will be low (Straub et al., 2005). Similarly, within vertebrates almost all ferric iron is sequestered by proteins such as lactoferrins, serum transferrins, hemoglobin or stored as ferritins in the cells. *Salmonella* therefore secretes high affinity molecules called siderophores to hijack ferric iron from complexed substances as well as to scavenge labile Fe^3+^ pool. The major iron sequestering component synthesized by all *Salmonella* species is enterobactin, a catecholate type siderophore. The proteins encoded by the *entABCDEF* operon, synthesize and assemble three *N*-2,3-dihyroxybenzoyl-L-serines to accommodate Fe^3+^ with a formation constant (K_f)_ of ∼10^49^ in a series of reactions (Scarrow et al., 1991; Figure 1B). There are no molecules in nature with matching affinity to iron like enterobactin, thus superior in chelating ferric iron. Apo enterobactin is released from the inner membrane via EntS and secreted via TolC residing in the outer membrane (Furrer et al., 2002; Bleuel et al., 2005). Outside the bacterial cell the ferric loaded siderophore interacts with the receptors FepA and/or IroN in the outer membrane to be internalized (Rabsch et al., 1999). These receptors form a barrel shaped channel with extracellular loops for interaction with the siderophore (Figure 1A). A cork (plug) domain lies embedded within FepA/IroN to occlude the passage of molecules at the resting stage. The energy and force generated by the ExbB-ExbD protein complex in the inner-membrane is mechanically transduced to the receptor via TonB. This mechanical force is believed to cause a conformational change in FepA or IroN, which moves the cork domain alleviating the blockage to internalize -Fe^3+^-enterobactin into the periplasm. The ferric-enterobactin complex is then shuttled via an ABC transporter through the inner membrane into the cytoplasm, where the iron can be extracted and utilized (mechanism has been reviewed by Köster, 2001; Krewulak and Vogel, 2008). Enterobactin can be further glycosylated to form salmochelin (via glucosyltransferase encoded by *iroB*) to evade lipocalin-2 mediated sequestration of ferric-enterobactin mounted by vertebrate hosts during infection. Lipocalin-2 (neutrophil gelatinase-associated lipocalin/LCN2/Lcn2) is an important antimicrobial component secreted from neutrophils and other immune cells. It binds with high affinity to the ferri-enterobactin complex (but not the ferric iron), depleting iron for *Salmonella* (Goetz et al., 2002). Raffatellu et al. (2009) showed that salmochelin synthesis and uptake provided a competitive advantage for replication and survival of STm in the inflamed intestines of mice due to the fact that it provided resistance to Lcn2. Some studies revealed that catecholate siderophore synthesis is not required to cause mouse typhoid indicating that requirement of iron during growth in extraintestinal sites might differ from the intestinal milieu in murine models (Benjamin et al., 1985; Tsolis et al., 1996; Cunrath and Bumann, 2019). Oral supplementation of iron in mice with iron deficiency anemia increased the iron saturated transferrin fraction and led to overgrowth of STm in the liver and spleen (Hoffmann et al., 2021). This overgrowth of STm was directly related to the ability to acquire iron mediated partly by catecholate siderophores (enterobactin, salmochelin) and FeoABC. Overall, data suggested the functional redundancy of the high affinity iron acquisition systems of STm in certain conditions, such as an imbalance of host’s iron homeostasis (Hoffmann et al., 2021). All the evidence above suggests that there is a molecular arms race for iron between pathogens and the host which can decide the clinical outcome. The question remains whether this same arms race occurs in chickens during *Salmonella* infection and colonization.

There are several reasons why it is highly important to investigate the role of iron homeostasis of *Salmonella enetrica* serovar enteritidis (SEn) in a chicken model. Chicken (*Gallus gallus domesticus*) acts as a reservoir host for SEn which accounts for most human epidemics globally. Handling, consumption of contaminated meat or egg products trace back to most outbreaks associated with SEn and other non-typhoidal *Salmonella* serovars (NTS) (Wellawa et al., 2020). Therefore, understanding the role of iron uptake systems in virulence will help to utilize iron-regulated gene clusters as novel therapeutic targets against chicken associated NTS to minimize colonization of birds. For example, siderophore receptors have been used as promising vaccine candidates in experimental models and in some commercial vaccines to protect against bacterial infections in livestock (Hermesch et al., 2008; Kaneshige et al., 2009; Thornton et al., 2009; Gorden et al., 2018). So far, our insights into the role of iron uptake systems of NTS is based on STm infection using murine models. It is difficult to extrapolate data from murine models to the chicken largely due to differences in the immune systems (Wigley, 2014). Here we have analyzed the competitive advantage of being able to utilize high affinity iron acquisition of SEn during cecal and systemic colonization in a chicken model. Birds were co-infected with a wildtype SEn and a mutant strain carrying deletions in iron regulated gene clusters. In co-infection experiments each animal acted as a control of its own thus the so-called “pen effect” is avoided. Our approach was to tag wildtype or mutant strains of SEn with a bioluminescent marker *luxCDABE* to facilitate tracking them while inside the bird and at the same time the marker allowed us to differentiate wildtype from mutant colonies (or vice versa) after direct plating on selective growth media followed by imaging. Further, in a second construct, a molecular switch was introduced upstream of the *lux* operon to assess the bioavailability of iron in the cecal compartment during early infection of day-old birds by imaging. Our data indicated that the two iron uptake strategies provided a competitive advantage for efficient colonization and redundancy during pathogenesis.

## Results

### Siderophore Synthesis and FeoABC Mediated Fe^2+^ Uptake Synergistically Provides a Fitness Advantage in Colonizing the Cecum of Chickens

EntB is a dual functioning protein (Gehring et al., 1997). The N-terminal region is known to have isochorismatase activity, which catalyzes the synthesis of 2,3-Dihydroxy-benzoate (2,3 DHB) (Figure 1B). In addition, EntB plays an important role in peptide bond formation between L-serine amino acids and 2,3 DHB to give rise to the final architecture of enterobactin (Reitz et al., 2017). Therefore, deletion of *entB* completely abolishes the catecholate siderophore production. This was evident through lack of yellow halo formation by SEn colonies grown on chrome azurol agar plates (Figure 1B). To investigate the role of siderophore production in colonization, 1-week old commercial broilers were challenged by co-infection with SEn Δ*entB* and an isogenic SEn WT strain. SEn strains were tagged with the bacterial luciferase operon (*luxCDABE)* inserted at the *attTn7* site in the chromosome and controlled by a synthetic, sigma 70-dependent promoter (sig70c10; Shivak et al., 2016). For the convenience of use, we designated as “c10 lux” in this study. This technique has been previously utilized to track STm infection in mice *in vivo* and *ex vivo* (Shivak et al., 2016). In our experiments, we tracked light production using a bioluminescent imager (IVIS Lumina II) and used chloramphenicol to differentiate tagged SEn strains from other bacterial species that can grow on brilliant green agar plates (i.e., *Escherichia coli*, *Proteus*, *Pseudomonas*, and *Citrobacter* sp.).

**FIGURE 2:**
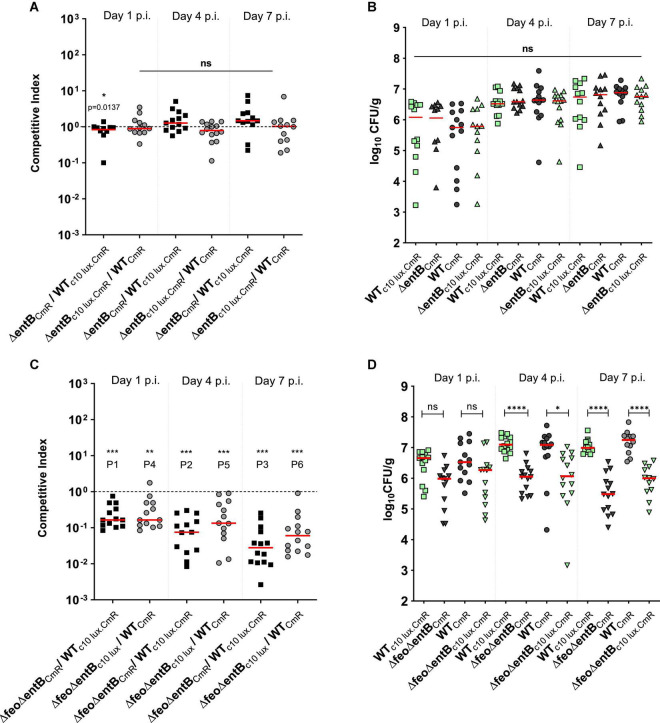
**(A,C)** competitive index scores; mutant vswildtype in cecal tissue. A; *ΔentB* vs wildtype strain, C; *ΔfeoΔentB* vs wildtype strain. Each symbol represents a CI value per bird (12–14 commercial broilers/group) calculated based upon bacteria recovered from cecal content as presented in figure B and D respectively. Median value of the data is given by the horizontal bar. Dotted horizontal line is the CI value of 1 which indicates that the mutant is equally competitive as the wildtype strain. ^***^P1, ^***^P2 = 0.0002, ^***^P3 = 0.0001, ^**^P4 = 0.0034, ^***^P5 = 0.0002, ^***^P6 = 0.0001. **(B,D)** Bacterial load in the cecal content. Each symbol represents a colony count of a particular bird, and each bird has a matching pair of wildtype and mutant colony counts due to the co-infection. The detection limit of *Salmonella* colonies in the brilliant green agar plate is around 100 CFU/g. Mean differences were calculated by appropriate statistical test. ns; no significance. *p = 0.0142, ^****^p ≤ 0.0001.

We performed two co-infection experiments in parallel by switching the bioluminescent marker between the wildtype and the *entB* mutant strain to assess the fitness burden due to the presence of the luciferase reporter. Calculated competitive index (CI) scores for cecal colonization were not significantly different from the ratio 1 at all-time points examined (Figure 2A), meaning that the Δ*entB* strain was equally competitive as the wildtype strain of SEn in colonizing the cecum. CI is the ratio between ratio of output strains (mutant: wildtype) to ratio of inoculum strains (mutant: wildtype) (see section “Materials and Methods” for the equation). This data was consistent for Δ*entB* expressing *lux* (Δ*entB*_c10lux.CmR_) as well as the mutant strain without the bioluminescent marker (Δ*entB*_CmR_) indicating that luciferase expression under promoter sig70c10 had no significant cost during colonization of the cecal environment (Figure 2A). One exception was that on day 1 post infection (p.i.) WT_c10lux.CmR_ marginally outcompeted the Δ*entB*_CmR_ (Figure 2A) resulting in a statistical difference (*p* = 0.0137), but this was not observed in a similar manner in the other group: Δ*entB*_c10lux.CmR_ versus WT_CmR_ (Figure 2A). Lack of repeatability of this observation makes a strong argument that it might be biologically insignificant. The overall median bacterial colony count was ∼10^6^ CFU/g of cecal content at the beginning (day 1 p.i.) and increased by 0.5 log by day 7 p.i. for both the mutant and the wildtype strain (Figure 2B). This suggested that after early implantation in the cecal environment, the SEn population remained at a rather constant level during our experimental challenge model with this SEn strain. Our finding suggested that the loss of catecholate siderophore production did not cause a significant fitness defect in colonizing the cecum and survival until day 7 p.i. in 1-week old commercial broilers.

We created a double knockout strain (Δ*feoΔentB*) affecting both ferric and ferrous high affinity iron acquisition and performed another series of co-infections. The wildtype strain outcompeted the Δ*feoΔentB* strain at all time points examined (Figure 2C). The Δ*feoΔentB* strain was impaired in cecal colonization (Figure 2C), with median CFU values > 0.5 log less than the wildtype strain at day 1 post infection increasing to greater than 1 log lower by day 7 (Figure 2D). While the population of the wildtype strain expanded from ∼10^6^ CFU/g on day 1 p.i. to ∼10^7^ CFU/g on day 7 p.i., the SEn strain lacking high affinity iron uptake decreased toward median values of ∼10^5^ CFU/g by day 7 p.i. (Figure 2D). These results indicated that the Δ*feoΔentB* strain was not able to colonize the cecum efficiently in the presence of a competitor for iron like wildtype SEn.

**FIGURE 3:**
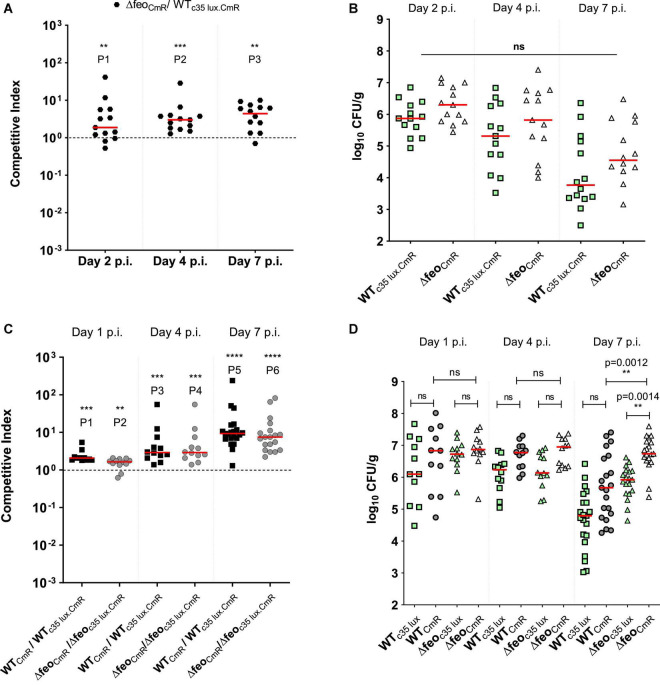
**(A)** Competitive index scores; Δ*feoABC* with chloramphenicol resistant marker (Δ*feo*_*CmR*_) vs. bioluminescent wildtype SEn strain in cecal tissue. Thirteen broilers/group. Each symbol represents CI value obtained from a bird. ^**^P1 = 0.0081, ^***^P2 = 0.0002, ^**^P3 = 0.0012. **(B,D)** Bacterial load in the cecal content. Each symbol represents a colony count of a particular bird, for each strain. Mean differences were analyzed using appropriate statistic test. ns, not significant. **(C)** Competitive indexes for non-bioluminescent strain vs. bioluminescent strain. ^***^P1 = 0.001, ^**^P2 = 0.0034, ^***^P3, ^***^P4 = 0.0005, ^****^P5, ^****^P6 ≤ 0.0001.

To assess the impact of the high affinity ferrous iron uptake system, we performed a co-infection between the wildtype WT_c35lux.CmR_ and the Δ*feo*_*CmR*_ strain in 1-week old commercial broilers. WT_c35lux.CmR_ carried the high expression of the *luxCDABE* operon under control of the sig70c35 promoter (designated as the “c35 lux” in this study). Calculated CI values for cecum suggested that the Δ*feo*_CmR_ strain outcompeted the WT_c35__lux.CmR_ strain (Figure 3A). Median CI values were ∼2 on day 2 p.i., ∼3 on day 4 p.i., and ∼4 at day 7 p.i. (Figure 3A). In other words, Δ*feo*_CmR_ strain maintained relatively higher numbers than its WT_c35lux.CmR_ at all time points examined (Figure 3B). However, the overall mean level of colonization did not statistically differ between the WT_c35lux.CmR_ and Δ*feo* (Figure 3B). Our results indicated that “c35 lux” marker constitutes a slight burden to the strain during cecal colonization but did not hamper the overall colonization ability in the cecum. This was further confirmed by a separate co-infection experiment conducted, where each group of birds were orally gavage with a tagged (c35 lux) and untagged strain of SEn with identical genetic backgrounds. CI was calculated between non-bioluminescent versus bioluminescent strains. As expected, non-bioluminescent strains; wildtype or Δ*feo* outcompeted the bioluminescent strain as reflected by the CI values (Figure 3C). Median CI values in the wildtype co-infected group were ∼2 on day 2 p.i., ∼3 on day 4 p.i. and ∼9 on day 7 p.i (Figure 3C). Birds infected with Δ*feo* strains, followed similar values except for on day 7 p.i. when the median CI was ∼8 (Figure 3C). This trend in gained competitiveness of the non-bioluminescent strain has been the reason for Δ*feo*_CmR_ to outcompete the WT_c35lux.CmR_ in the aforementioned experiment, which resulted in a similar trend of CI values (Figure 3A). However, the mean level of colonization between the bioluminescent strain and non-bioluminescent strain, showed no significant difference at day 1 and 4 p.i. (Figure 3D). The same trend was seen at day 7 p.i. except for the Δ*feo* infected group, where Δ*feo*_CmR_ outcompeted the Δ*feo*_c35lux.CmR_ (Figure 3D). Also, when similar tagged strains were compared, WT_CmR_ and Δ*feo*_CmR_ maintained a comparable mean level of colonization (∼10^7^ CFU/g) on day 1, 4 p.i (Figure 3D). Further, Δ*feo*_CmR_ maintained a trend of a relatively high mean colonization level at day 7 p.i. compared to WT_CmR._ All this indicated that the loss of Feo mediated Fe^2+^ uptake alone was not sufficient to cause a significant disadvantage in competing in the cecal environment.

### Siderophore Biosynthesis Provides a Competitive Advantage in Rapid Systemic Spread and Survival in Extraintestinal Sites

**FIGURE 4:**
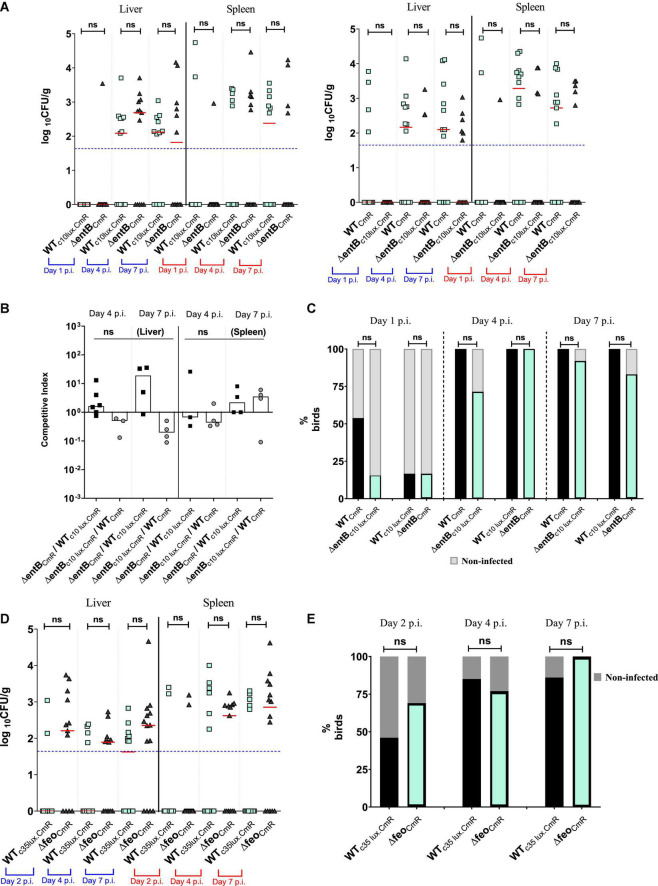
**(A)** Wildtype or Δ*entB* burden in liver and spleen by direct plating. Each symbol represents a log_10_ CFU value related to a mutant or wildtype strain. Due to co-infection each bird (12–14 birds per time point) has two matching pair values of CFU not shown in here. Median value of the data set shown by horizontal bar. Detection limit has shown by dotted horizontal line (50 CFU/g). Mean differences were accounted for analysis. ns, not significant. **(B)** Competitive index scores for Δ*entB* vs. wildtype SEn strain. CI values were calculated only from sample which showed recovery from direct plating. Column indicates the median value for the data set. **(C)** Infectivity of wildtype or Δ*entB* obtained by liver and spleen enrichments. Dark column represents % birds for infected by the strain which are recovered by liver/spleen enrichments. Non-infected % of birds presented by shaded gray color columns means that strain was not recovered by enrichments. **(D)** Wildtype or Δ*feo* burden in liver and spleen by direct plating. Each symbol represents a log_10_ CFU value related to a mutant or wildtype strain (13–14 birds per time point). Description of data presentation follows as **(A)**. **(E)** Infectivity of wildtype or Δ*feo* obtained by liver and spleen enrichments. Description of data presentation follows as **(C)**.

*Salmonella* Enteritidis is known to cause acute systemic infection in chicken and survive in internal organs such as liver, spleen, ovaries, and oviducts (Gast and Beard, 1990; Barrow, 1991). Here we were able to quantify the SEn burden in liver and spleen after oral inoculum of SEn carrying mutations affecting high-affinity, iron uptake. Liver and spleen colonization levels described in Figure 4A, represent data obtained from the same birds utilized in the experiment described in Figures 2A,B. There was no statistical significance in the mean level of colonization of Δ*entB* compared to the WT at all time points by direct plating onto XLD agar (Figure 4A). This was true for strains with or without c10 *lux* marker (Figure 4A). It was evident that wildtype SEn strains did not result in a good recovery (at least one colony) from liver and spleen by direct plating, even though birds were challenged with a high oral challenge dose of 10^9^ CFU per bird. Most samples were below the detection limit on Xylose Lysine Deoxycholate (XLD) agar plates (Figure 4A). CI scores for liver and spleen infectivity were calculated from samples which had both wildtype and mutant colonies (Figure 4B). Calculated CI values between the Δ*entB* and wildtype SEn were not significantly different from the ratio of 1, meaning both strains were equally competent in colonizing the liver and spleen (Figure 4A). However, enrichment data showed some important differences in the kinetics of systemic infection (Figure 4C). On day 1 p.i., ∼54% of the birds (7/13 birds) had systemic infection by WT_CmR_. That percentage was ∼17% (2/12 birds) when the wildtype strain was tagged with c10 lux (WT_c10lux.CmR_). In comparison to the wildtype strain, only ∼17% of the birds were infected by Δ*entB*_CmR_ while Δ*entB*_c10__lux.CmR_ had a similar level of infection (∼15%). The siderophore null strain was not able to reach systemic infectivity beyond 17% in both co-infection groups whether or not they carried the reporter (Figure 4C). This indicated that strains devoid of catecholate siderophore production showed slow progression into “systemic sites” such as liver and spleen. WT_c10lux.CmR_ was not able to present the same level of infectivity as observed in the WT_CmR_, co-infected group in this particular experiment. It was an indication that individual variation among birds contributes to different kinetics during seeding of *Salmonella* in birds (Figure 4C). Hence our approach to perform two parallel experiments was superior to alleviate those variations. After the initial lag phase, recovery of Δ*entB*_CmR_ from systemic sites reached 100% on day 4 p.i., which is comparable to WT_c10lux.CmR_ (Figure 4C). The same trend was observed in the group of birds co-challenged with Δ*entB*_c10lux.CmR_ (∼71%) and wildtype_CmR_ (100%). There was no significant difference in the ability to cause systemic infection at day 4 and day 7 p.i. (Figure 4C) by a SEn lacking siderophore synthesis. However, Δ*entB* showed a trend of reduction in recovering from enriched splenic compartment, compared to the wildtype SEn at day 4 post infection (Supplementary Figure 1). The average ability to recover from spleen at day 4 p.i. by Δ*entB* strain was 44.25% (average of the lux tagged and untagged). In comparison, the wildtype strain was able to infect 85.5% of spleens sampled at day 4 p.i. On day 7 p.i. the Δ*entB* strain showed 47.5% ability (average) to infect spleen while wildtype SEn maintained a 83.5% infectivity. The average infectivity of Δ*entB* at day 4 p.i. in liver was 78.5% and was 84% on day 7 p.i (Supplementary Figure 1). So relatively much higher than what was observed from spleen. Our data suggested that requirement of enterobactin or salmochelin might vary with different niches that *Salmonella* may encounter.

**FIGURE 5:**
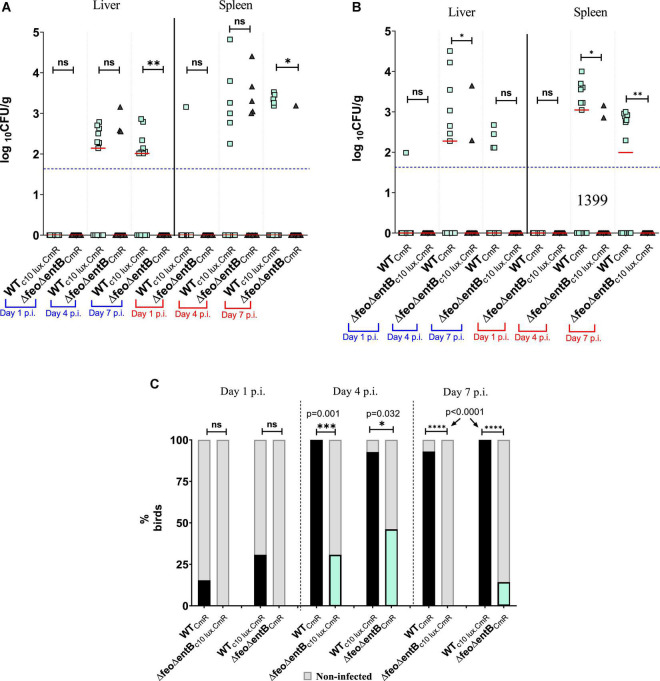
**(A,B)** Wildtype or Δ*feoΔentB* burden in liver and spleen by direct plating. Each symbol represents a colony count of a particular bird (13–14 birds/time point), and each bird has a matching pair of wildtype/mutant colony counts due to the co-infection. Median value of the data is given by the horizontal bar. The detection limit of the XLD agar plate is around 50 CFU/g as indicated in horizontal dotted line. Differences between the median of the data set was accounted. In **(A)** *, *p* = 0.0407 (spleen), ^**^p = 0.0019 (liver); **(B)** *p = 0.0492 (liver), *p = 0.0159 (spleen), ^**^, *p* = 0.0058 (spleen). **(C)** Infectivity of wildtype or Δ*feoΔentB* obtained by liver and spleen enrichments. Dark column represents % birds for infected by the strain which are recovered by liver/spleen enrichments. Non-infected% of birds presented by shaded gray. Fisher’s exact test used for statistical analysis. ns: not significant. ^***^p = 0.001, *p = 0.032, ^****^ *p* = <0.0001.

The loss of Feo-mediated Fe^2+^ uptake alone, did not show a significant competitive disadvantage in the ability to cause systemic infection or progression into systemic sites according to the data obtained from birds co-challenged with Δ*feo*_CmR_/WT_c35lux.CmR_ (Figures 4D,E). The overall mean level of colonization in liver and spleen by Δ*feo*_CmR_ did not statistically differ compared to the WT_c35lux.CmR_ at all times examined (Figure 4D). At day 2 p.i., the Δ*feo*_CmR_ strain was able to infect ∼69% of the birds examined while the WT_c35lux.CmR_ strain was recovered from ∼46% of the birds (Figure 4E). We were not able to sample on day 1 p.i. in this experiment. However, in a separate experiment, birds co-infected with Δ*feo*_CmR_/Δ*feo*_c35lux.CmR_, Δ*feo*_CmR_ were able to reach 50% systemic infection on day 1 p.i while Δ*feo*_c35lux.CmR_ reached ∼42% (Supplementary Figure 2). In parallel, systemic infectivity of WT_CmR_ was ∼73% on day 1 p.i. and for WT_c35lux.CmR_, it was ∼36% (Supplementary Figure 2). Together with this data, we demonstrated that loss of *feo* alone did not impair the ability of SEn in rapid systemic spread. This result is in contrast to the loss of enterobactin synthesis alone, which relatively decreased the rapid systemic spread at day 1 p.i (Figure 4C). Even though *feo* deletion did not show any burden in the systemic infection, loss of both *feo* and *entB* showed a much greater magnitude of reduction in the ability to cause systemic infection and colonization in the birds (Figure 5). The median colony counts for Δ*feoΔentB* obtained by direct plating from liver and spleen tissues were below the detection limit at all times points examined (Figures 5A,B). This was true for Δ*feoΔentB*_CmR_ and Δ*feoΔentB*_c10lux.CmR_ (Figures 5A,B). Consistent with previous experiments, recovery of wildtype SEn by direct plating either spleen or liver remained intrinsically low where most birds had numbers of bacteria below the detection limit (Figures 5A,B). However, compared to the WT_c10lux.CmR_, Δ*feoΔentB*_CmR_ had a significantly low median level of colonization on day 7 p.i. in both liver and splenic compartment (Figure 5A). Similarly, compared to the WT_CmR,_ the median colonization level of Δ*feoΔentB*_c10lux.CmR_ strain in liver and spleen was significantly low on day 4 p.i. (Figure 5B). Also, Δ*feoΔentB*_c10lux.CmR_ resulted in a significant reduction in median colony counts on day 7 p.i. in splenic tissue except for the liver (Figure 5B). Overall, our data indicated a statistically significant drop in the extraintestinal colonization level when both ferrous iron uptake and siderophore mediated ferric iron uptake is lost during colonization in liver and spleen. This observation was further confirmed by enrichments of liver and spleen tissue homogenates (Figure 5C). Enrichment of liver and spleen tissues were not able to recover any of the Δ*feoΔentB* strains at day 1 p.i. compared to wild type strains. The average ability to cause systemic infection by a strain devoid of high affinity iron acquisition systems was 38.5% (Δ*feoΔentB* with or without c10*lux*) on day 4 post infection. This is in contrast to the data obtained from birds co-challenged with Δ*entB* and Δ*feo* alone where it reached 100% in some instances (Figures 4C,E). Survivability of Δ*feoΔentB*_CmR_ in liver/spleen significantly dropped from ∼46% at day 4 p.i. to ∼14% on day 7 p.i. (Figure 5C). A similar trend was observed for Δ*feoΔentB*_c10__lux.CmR_ for the same time points; ∼31 to 0%. In contrast, wildtype strains (with or without c10 lux) maintained close to 100% systemic infectivity on day 4,7 p.i (Figure 5C).

### High Affinity Iron Uptake Systems Are Important for Survivability of *Salmonella* Enteritidis in Chicken Macrophage -Like Cells

**FIGURE 6:**
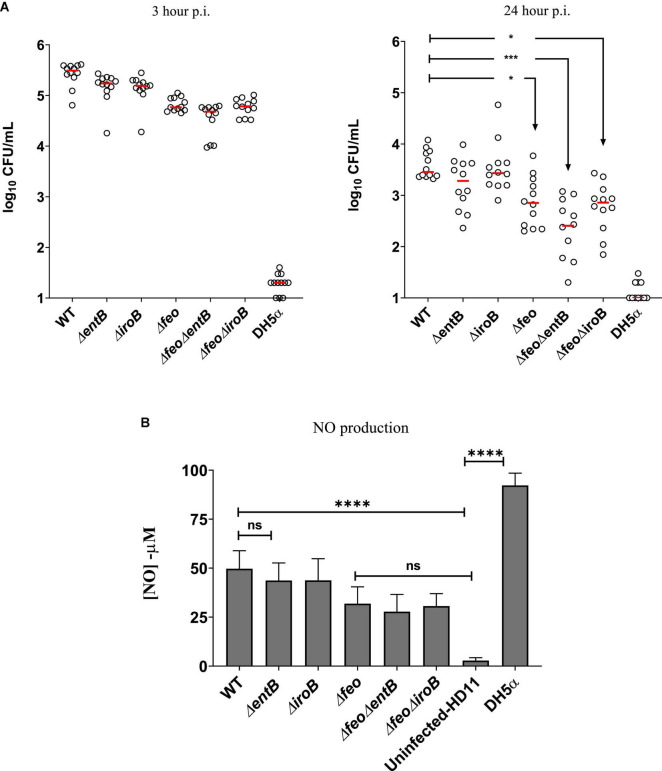
**(A)** Gentamycin protection assay results at 3 and 24 h of post infection. Survived bacteria shown as CFU/ml in log10 scale. Assay was performed four times in three technical replicates for each strain. Each symbol represents one technical replicate. The detection limit of LB agar palate is 10 CFU/ml. Horizontal bar represent the median value. Mean differences was analyzed using log10 values of CFU/ml. *p = 0.028, *p = 0.012, ^***^p = 0.0003. **(B)** Nitric oxide (NO) concentration in μM, 24 post infection. Each column represent mean with standard deviation. Data was from three assays run in triplicates. Statistical significance represents mean differences. ns, not significant, ^****^p ≤ 0.0001.

The macrophage is one of the major phagocytic cells where *Salmonella* can infect, replicate and survive during trafficking to extraintestinal sites (Haraga et al., 2008; Watson and Holden, 2010). We performed a gentamicin protection assay utilizing chicken macrophage-like HD-11 cells to determine whether survivability of SEn in chicken macrophages can be affected by deletion of high affinity iron uptake systems. Our results indicated that after 24 h, the deletion of *feo* alone resulted in a significantly lower survivability of the mutant strain compared to the wildtype strain, and survival was further decreased by the lack of siderophore production in a Δ*feoΔentB* strain (Figure 6A). In contrast to the Δ*feoΔentB*, the Δ*feoΔiroB* which specifically lacks salmochelin production showed a similar level of survivability compared to the Δ*feo* alone. In other words, salmochelin production may not be necessary during initial stages of survival inside chicken macrophages. However, lack of siderophore synthesis alone did not show a significant difference in the survivability at 24-h p.i (Figure 6A). It is plausible that SEn may preferentially uptake Fe^2+^ under *in vitro* culture conditions. Intracellular wildtype SEn counts dropped from ∼10^5^ CFU/mL on 3-h p.i. to ∼10^3^CFU/mL on 24-h p.i., which suggested that SEn might not cause an efficient systemic infection in birds. In line with our *in vitro* findings, we saw that recovery of wildtype SEn in orally challenged birds was mostly below the detection limit as shown after direct plating on XLD agar (Figures 4A,D, 5A,B). Even though Δ*feo* alone resulted in some degree of reduction in the survivability at 24 h post infections, this effect might be neglectable, since the wildtype strain is not a good systemic survivor. Hence, the most prominent impact on the survivability of SEn in macrophages is reflected only by the Δ*feoΔentB* strain (*p* = 0.0003, ^***^). The percentage of live HD11-cells remained comparable (average 65%) in SEn infected groups at 24 h p.i. during intracellular survival (Supplementary Figure 3). Compared to the SEn strain, the *E coli* DH5α strain survived poorly and accompanied a high amount of macrophage cell death (89%) after 24 h of infection (Supplementary Figure 3). It is well known that some of the effectors secreted through the type III secretion system encoded by *Salmonella* Pathogenicity Island 2 (T3SS2) can delay necroptosis (Jennings et al., 2017). Since *E coli* DH5α lacks similar specialized systems to inhibit cell death, it was not surprising to see a significant number of dead cells after infection. In parallel, there was a significant increase of reactive nitrogen species (means of NO production) generation at 24 h from DH5α-infected macrophages cells (>90 μM) compared to SEn-infected cells (Figure 6B). Previous experiments have shown that infection of HD-11 cells with heat killed strains of SEn significantly increased the NO generation reaching > 100 μM at 24 post infections but not with live cells (He et al., 2012). One possible explanation for such observations including in this study, may be that SEn encodes a number of detoxification enzymes (ex: flavohemoproteins) to reduce the nitric oxide production in the cell (Henard and Vázquez-Torres, 2011) while DH5α lacks the same arsenal of mechanisms. SEn strains also showed a relatively high amount of NO generation (measurement of RNS) compared to the un-infected HD11 cells reaching up to a ∼50 μM (mean value) by the wildtype strain-infected cells. Mutants carrying the *feo* deletion showed relatively low amount of NO production (∼27–31 μM) compared to the wildtype. NO production in *Salmonella* infected HD11 cells may correlate with the amount of viable intra-macrophage *Salmonella*. For example, live intra-macrophage wildtype *Salmonella* is higher than mutants carrying *feo* deletions, and similarly NO production is relatively the highest in wildtype infected cells rather than in mutants with *feo* deletions. A correlation between the amount of viable SEn strains to NO production in HD-11 cells has been reported elsewhere, hence our data is consistent with that information (He et al., 2013).

### *Salmonella* Enteritidis Experiences Iron Limiting Conditions During Colonization in the Cecum

**FIGURE 7:**
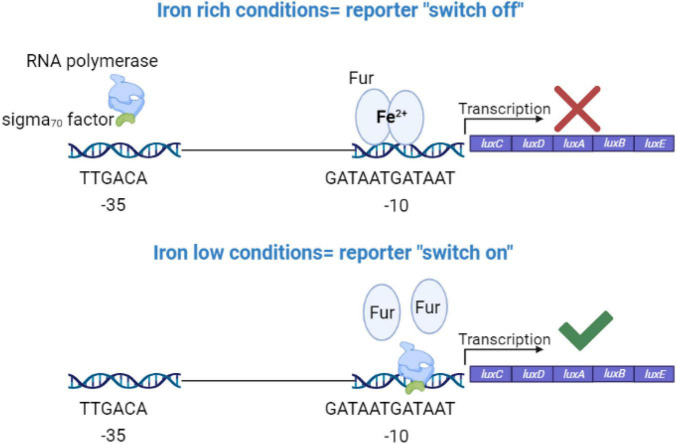
Molecular design for the iron sensing promoter region. TTGACA sequence is recognized by sigma 70 factor which directs the RNA polymerase to the promoter region. GATAATGATAAT is the consensus sequence recognized by ferric uptake regulator protein (Fur) and known as the fur box. In *Salmonella*, ferrous iron together with Fur, form a dimer and bind to fur box like sequences upstream of iron regulated genes to block the transcription under iron sufficient conditions.

**FIGURE 8:**
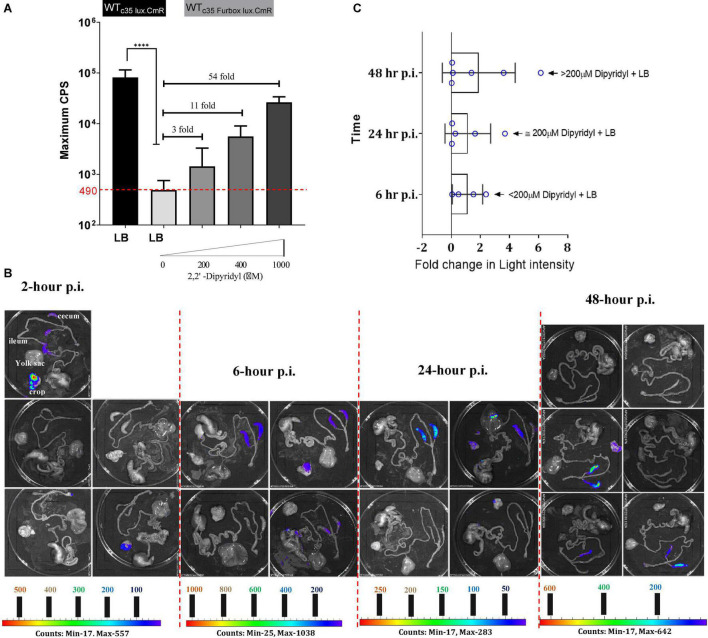
**(A)** Fur box reporter (WT_*c*35Furbox lux.CmR_) expression under iron replete and iron deplete conditions. Maximum light intensity was obtained from luciferase assay results from 24 biological replicates run by a plate reader. 2,2′ Dipyridyl (DP) used with varying concentrations to chelate iron in the LB. Fold change is calculated relative to the average value of reporter expression by the WT_c35Furbox lux_. Median of the data (CPS) was compared for statistics. ^****^P ≤ 0.0001. **(B)** Bioluminescent imaging of gastrointestinal tract of SPF birds challenged with reporter WT_c35Furbox lux.CmR_). Lowest to highest signal intensity is shown in rainbow color scale. Each pixel represents a specific signal intensity. All images were taken at the IVIS settings of; *f* = 1, binning = medium, exposure = 60 s, filter = open. **(C)** Estimated Fur-mediated de-repression in the cecum as a fold change in light intensity. Fold change was calculated from dividing assigned light intensity for each cecal compartment, by 490 CPS. Maximum iron starvation at each time point has been indicated by means of iron chelation of in LB using dipyridyl.

Ferric uptake regulator (Fur) is considered as the global transcriptional regulator in iron homeostasis in many bacteria including SEn (Hantke, 1981; Bagg and Neilands, 1987; Hassan and Troxell, 2013). Fur protein (17–21 kDa) binds to Fe^2+^ in the bacterial cell and undergoes a conformational change (dimerization). The Fur-Fe^2+^ dimer recognizes a sequence identical to or highly resembling the “Fur box” (GATTATGATTAT) upstream of many iron regulated genes overlapping typical promoter elements (Figure 7). Under iron replete conditions Fur-Fe^2+^, binds to the Fur box and blocks (represses) the transcription of downstream located genes. De-repression will happen under iron-deplete conditions. Here we designed an iron sensitive, Fur regulated, promoter (sigma70) by cloning the Fur box sequence upstream of a *luxCDABE* (at -10 region) so it can block the sigma 70 factor driven RNA polymerase reading 35 bp upstream of transcription initiation site (Figure 7). Finally, this “molecular switch” construct was introduced into the chromosome of SEn at the *attTn7* site (WT_c35Furbox lux.CmR_). As we expected, when WT_c35Furbox lux.CmR_ was grown in iron rich LB medium, expression of *luxCDABE* (measured by counts per second of emitted signal) was strongly repressed compared to the WT_c35lux.CmR_ strain which didn’t have the Fur box (Figure 8A). WT_c35lux.CmR_ showed a maximum mean value of 80,000 CPS while WT_c35Furbox lux.CmR_ only reached a maximum of 490 CPS. Addition of an iron chelator (2,2′-dipyridyl) to the LB medium caused a dose dependent de-repression of *luxCDABE* (Figure 8A). One-day old specific pathogen free chickens (21 birds) were orally gavaged receiving WT_c35Furbox lux.CmR_ strains. *Ex vivo* imaging was then performed to detect the kinetics of Fur-mediated de-repression during early establishment of the bacteria in the cecum (2-, 6-, 24-, and 48-h post infection). In parallel, another 21 birds were challenged with WT_c35lux.CmR_ which constitutively expressed the *luxCDABE* under sig70c35 promoter (Shivak et al., 2016) without the Fur box mediated barrier. Here we utilized the WT_c35lux.CmR_ strain as a tool,

(I) for the detection of SEn in the gastrointestinal track by imaging. It has the advantage of emitting a strong bioluminescent signal when the bacterial cells are replicating and alive;
(II) to analyze the degree of Fur mediated de-repression visually and quantitatively relative to the WT_c35lux.CmR_.

*Salmonella* Enteritidis carrying each reporter able to colonize the chicken cecum in an identical manner (Supplementary Figure 4B). The median SEn concentration at 2 h p.i. was ∼10^6^ CFU/g and reached to a ∼10^8^ CFU/g 6-h p.i. (Supplementary Figure 4B). SEn concentration was maximized to ∼10^9^ CFU/g at 24-h p.i. It remained in the same range 48-h p.i. (Supplementary Figure 4B). This kinetic of bacterial recovery from cecal content highly suggested that actual colonization started after 24-h of infection, where SEn managed to maintain a stable population within the cecal environment. Two hours post infection, most birds infected with WT_c35Furbox lux.CmR_ strain, did not result in a detectable bioluminescent signal from the cecum (Figure 8B). Maximum signal intensities were consisted with areas of 17 photon counts in the cecum which was detectable in one bird (Figure 8B). Undetectable signals by imaging may have resulted from low signal strength (de-repression) generated by the strain WT_c35Furbox lux.CmR_. The detection limit also depended on the bacterial concentration which can generate a certain threshold of signal hence ∼10^6^ CFU/g might not have been enough to generate detectable signals. Signal intensities improved from 24-h p.i. to 48-h p.i. with areas having maximum of 400–600 photon counts (Figure 8B). This increment of signal strength is solely indicative of true Fur mediated de-repression of *lux* expression by the strain since the bacterial concentration remained similar at 24− and 48-h p.i. (Supplementary Figure 4B). In comparison to birds infected with WT_c35Furbox lux.CmR_ strain, birds infected with WT_c35lux.CmR_ showed visually contrasting images from 6 h post infection with areas having > 400 photon counts and reached maximum of ∼16,000 counts as represented by blue-green color in the scale (Supplementary Figure 4A). This is not surprising because *lux* expression in WT_c35lux.CmR_ was not under Fur-mediated transcriptional control. Our imaging data clearly indicated that bioavailability of iron to SEn, might not be extremely low during early cecal colonization events in young birds. If it was extremely low, the de-repression of Fur in WTc_35Furbox lux.CmR_ should have resulted in signal strength comparable to the level of WT_c35lux.CmR_. However, we saw a clear trend in the increment of bioluminescent signal strength toward 48-h p.i., suggesting that SEn must undergo a certain degree of Fur-mediated de-repression. We calculated the degree of Fur mediated de-repression of *lux* by accounting the radiant energy emitted from the surface of cecum (radiance = watt per steradian per square meter). We assumed that, under fully de-repressed status or strictly low iron environment, WT_c35Furbox lux.CmR_ strain should give rise to a maximum of 80,000 CPS value of light intensity comparable to WT_c35lux.CmR_ (Figure 8A). If WT_c35Furbox lux.CmR_ was fully de-repressed *in vivo*, radiance values calculated for the cecum compartment should be comparable to birds infected with WT_c35lux.CmR_. This rationale was used to estimate the amount of maximum signal strength which might give rise by the WT_c35Furbox lux.CmR_ in individual bird in a specific time point during colonization (see Supplementary Data File 1). The mean Fur mediated de-repression of bioluminescent signal remained < 2 fold for a 6–24-h p.i. and then increased to a twofold value at 48-h p.i. (Figure 8C).

## Discussion

The contribution of iron regulated genes of *Salmonella enterica* species to pathogenesis was vastly characterized using mammalian models. Among these, murine infection by STm has been the frequent *in vivo* model of interest. A murine typhoid model, akin to human typhoid fever, was the popular model to characterize virulence determinants during proliferation in liver and spleen. Mortality could be detected within 4–6 days in the typhoid model hence robust in terms of detecting attenuation. In most scenarios, murine typhoid models revealed that iron uptake utilizing catecholate siderophores and FeoABC transporters were not essential nor provided competitive advantage during acute systemic infections (Benjamin et al., 1985; Tsolis et al., 1996). A recent study conducted by Cunrath and Bumann (2019), confirmed that STm lacking both FeoABC mediated Fe^2+^ and TonB-dependent Fe^3+^ uptake (Δ*feoΔtonB*) was able to replicate in spleen without a fitness defect, in susceptible or resistant mice backgrounds during typhoid disease (Cunrath and Bumann, 2019). It is plausible that iron may not be limiting, or requirement of iron has been fulfilled by low affinity iron uptake systems, during replication in splenic or liver compartments. Increasing evidence suggested that requirement of Mn^2+^ exceeds that of iron during murine typhoid diseases (Janakiraman and Slauch, 2000; Boyer et al., 2002). Absolute requirement of the MntH and SitABCD cation transporters contributing to the virulence of STm was shown by Zaharik et al. (2004), in which all *Salmonella* resistant mice (Nramp^+/+^) survived an intraperitoneal challenge of a Δ*sitABCDΔmntH* mutant strain (Zaharik et al., 2004). Since MntH and SitABCD show higher affinity to Mn^2+^ compared to Fe^2+^, these results were more indicative that manganese and to a lesser extent iron is limiting during extraintestinal infections. Under certain conditions manganese can replace iron as a co-factor. Under stressed conditions *E. coli* demonstrated a resistance to oxidative damage using detoxification systems dependent solely on Mn^2+^ instead of Fe^2+^, by modulating the metabolism to a more manganese dependent manner (Sobota and Imlay, 2011). It is difficult to speculate about timing of this switch *in vivo*, but we know that natural resistance-associated macrophage protein 1 (Nramp1), recruited to the phagosomal membrane, can diminish Mn^2+^ more efficiently than Fe^2+^ from the *Salmonella* containing vacuole (SCV) (Jabado et al., 2000). In their findings, Cunrath and Bumann (2019) showed that the divalent metal transporter Nramp1 only caused a moderate level of iron deprivation during murine infection by STm (Cunrath and Bumann, 2019). Their findings argue that Nramp1 deprive more Mg^2+^ over iron, in the acute phase of infection in mice (Cunrath and Bumann, 2019). In this study, we provide evidence in favor of iron uptake via major facilitators, catecholate siderophores and FeoABC transporters, playing an important role during pathogenesis of SEn in a chicken.

In our oral gavage chicken model, lack of catecholate type siderophores alone, caused a low bacterial recovery rate from liver and spleen compared to the wild type SEn during initial stages of the infection (Figure 4C). In a duplicated experiment, a siderophore null (Δ*entB*) strain was only able to infect less than 20% of the birds examined (Figure 4C). It was further abrogated by the mutation in *feoABC*, in which Δ*feoΔentB* was not recovered by enrichments after 24 h of infection (Figure 5C). Hence, it is a strong indication that iron is limiting and both high affinity uptake mechanisms provide a competitive advantage during events taking place at the initial site of invasion. Since Δ*entB* alone caused a relatively greater reduction of systemic infectivity (∼15% – ∼17%) compared to Δ*feo* alone (∼50%) on day 1 p.i. (Supplementary Figure 2), our data strongly supported the idea that during rapid systemic dissemination Fe^3+^ uptake via catecholate siderophores will be critical. However, the bioavailability of the different forms of iron in various types of cells in the gastrointestinal tract of chicken is not known. In general, reductase enzymes convert Fe^3+^ to Fe^2+^ for uptake through the divalent metal transporters into the cytosol at the apical surface of enterocytes (Tako et al., 2010). The cytosolic fraction of iron serves as precursor for red blood cell generation after it has been transported by transferrin. The rest is bound by ferritins, iron-sulfur clusters, and metal complexing ligands (e.g., carboxylates, phosphates, amides, thiolates and hydroxylates). This pool of iron consisting of Fe^2+^/Fe^3+^, is considered exchangeable and chelatable at physiological conditions, hence known as labile cellular iron (LCI) (Cabantchik, 2014). Estimated LCI can be approximately 0.5–1.5 μM in human cells (<5% of total iron) which lies within the range of iron requirement of *Salmonella* for replication suggesting that LCI is vulnerable for exploitation by most bacteria (Kakhlon and Cabantchik, 2002). LCI is highly dynamic and variable in different type of cells (Kruszewski, 2003). Since our data indicate ferric iron is needed during rapid systemic dissemination, it is possible that a rich source of ferric iron might be present in the gut. We believe that Fe^3+^ sequesters, such as lactoferrin and tissue associated transferrin, will provide an accessible pool of ferric iron because these molecules are evidently upregulated during infection and stress conditions, respectively (Williams et al., 2006; Drago-Serrano et al., 2010). *Salmonella* is known to modulate vesicular trafficking enabling them to internalize the host’s nutrients via fusion of vesicles with *Salmonella* induced filament formation (Knodler and Steele-Mortimer, 2003). Thus, vesicles enriched with ferric, iron-bound ligands and proteins may provide an accessible pool of ferric iron in the vacuolar environment. In serum, the transferrin-bound Fe^3+^ fraction will be the predominate ionic form for the survival of SEn during the transient bacteremia phase. The bioavailability of free iron in the serum is estimated to be extremely low or undetectable in healthy mammals since almost all iron is bound by serum transferrin. Hence the secretion of high-affinity catecholate siderophores will be essential to resist iron starvation in the serum. Future experiments utilizing a specific mutation such as Δ*iroB* (glycosyltransferase) which blocks the glycosylation of enterobactin will provide more insight into the role of the “stealth siderophore” salmochelin, during bacteremia of SEn in chickens.

Similar to mammals, it is believed that the extraintestinal dissemination in the chickens depends on the ability of *Salmonella* to penetrate the intestinal epithelial cells (via trigger or zipper mechanisms) and to survive in the reticuloendothelial system (Bailey et al., 2005; Mastroeni et al., 2009). In chickens, heterophils are the major polymorphic nuclear cells which contribute to phagocytosis after bacterial invasion into epithelial cells. Heterophils are functionally equivalent to mammalian neutrophils but significantly differ in composition (Harmon, 1998). They are highly efficient in killing most of the *Salmonella* cells at the site of infection to provide disease resistance in chickens compared to mammals (Swaggerty et al., 2005). In fact, immunocompetency of certain chicken lines depended on a highly active population of heterophils which can be selected for disease resistance (Swaggerty et al., 2003). We speculate that catecholate siderophores are important to mediate resistance to heterophils at the gastrointestinal interface enabling a certain SEn population to systemically disseminate. The role of catecholate siderophores for the survival of intra-heterophilic *Salmonella* still needs to be published. Like intracellular lifestyle in macrophages, intra-heterophil *Salmonella* may need to overcome metal starvation mounted by Nramp-1. An adequate iron supply may ensure *Salmonella* to withstand the respiratory burst effect mounted by heterophils. In mammals, neutrophils secrete bacteriostatic molecules, like Lipocalin-2 (murine Lcn-2), to impede uptake of Fe^3+^-loaded enterobactin by STm during inflammation in the gut (Goetz et al., 2002; Raffatellu et al., 2009). Lcn2 specifically sequesters the apo- or ferric-enterobactin complex but not salmochelin (Fischbach et al., 2006). The chicken equivalent of Lcn2 is now identified as extracellular fatty acid binding protein (Ex-FABP) (Correnti et al., 2011). Ex-FABP has been evolved not only to capture enterobactin, but also to bind salmochelin derivatives (mono-glycosylated enterobactin/S1) which are not normally bound by mature Lcn2 (Correnti et al., 2011). Hence, it is potent in meditating a low ferric iron environment for *Salmonella* when a certain threshold is accomplished. Upregulation of Ex-FABP has been evident during SEn infection in the cecum of chicken and is known to be secreted from epithelial cells, macrophages and heterophils (Matulova et al., 2013). In this sense, synthesis of cyclic salmochelin derivatives (S4, S3) might provide a competitive advantage in rapid systemic dissemination and survival within the gut. To prove this hypothesis, modeling is warranted utilizing a transgenic chicken with allelic loss of Ex-FABP to analyze the competitiveness of Δ*entB* in Ex-FABP^–/–^ background (similar to the experiment conducted in reference; Raffatellu et al., 2009). Catecholate siderophore production by *Salmonella* might also be important for surviving within gut associated lymphoid tissues (GALT) in chickens. GALTs may provide a port of entry for dissemination into extraintestinal sites. It has similar characteristics to mammalian GALT but lacks draining lymph nodes (Befus et al., 1980). *Salmonella* localizes to Payer’s patches (small intestine) and cecum tonsils (ilio-cecal junction) for antigen presentation in chicken. Previously, Tsolis et al. (1996) showed that TonB-mediated ferric iron uptake provided a competitive advantage in colonizing GALT including Payer’s patches in mice but did not provide the same advantage for growth in liver and spleen (Tsolis et al., 1996). Thus, the iron requirement of *Salmonella* surviving within GALT might differ from that in organs such as liver and spleen. This needs to be revisited in our model to analyze the kinetics of colonization in Payer’s patches and cecal tonsils by various mutant backgrounds.

Regarding the iron supply of *Salmonella*, our data showed a substantial functional redundancy between the two high affinity iron uptake strategies during SEn colonization of the liver and spleen of chickens. Birds co-challenged with SEn carrying a mutation in *feo* or *entB*, showed no obvious defect in infecting organs such as liver and spleen at days 4 and 7 post challenge according to the data obtained by direct plating (Figures 4A,D) and enrichments (Figures 4C,E). The moment both high affinity iron uptake strategies were lost, the recovery of the Δ*feoΔentB* strain by enrichment cultivation showed significantly lower numbers at days 4 and 7 p.i. compared to the parent strain (Figure 5C). All these experiments were performed in duplicate with or without bioluminescent marker and the kinetics of colonization were similar on both occasions. Also, the median bacterial burden of Δ*feoΔentB* (CFU/g) as determined by direct plating using liver and spleen tissues, was maintained below the detection limit for the same consecutive days (Figures 5A,B). Especially in splenic tissue, we were able to see a statistically significant low burden of Δ*feoΔentB* strain on day 7 p.i. in both co-infections groups (Figures 5A,B). However, statistical significance was not repeated by liver tissue vastly due to variation in the wildtype colonization level in birds. It is speculated that most *Salmonella* may reside inside chicken macrophages at extraintestinal sites (Chappell et al., 2009; Roche et al., 2021). We measured the survivability of Δ*entB*, Δ*feo* and Δ*feoΔentB* strains of SEn in activated chicken macrophage like cells *in vitro*. The strongest defect in the survivability was observed by Δ*feoΔentB* (Figure 6A). This reflected that both high affinity iron uptake mechanisms synergistically provided a basis to overcome iron starvation in macrophages and this might have been one of the reasons for the colonization defect seen in extraintestinal organs such as liver and spleen from the double knockout strain. Our data also provided evidence for preferential uptake of iron through FeoABC and catecholate siderophores during colonization of SEn in liver and spleen. Hence our findings argue to the role of low affinity iron uptake via MntH and SitABCD in chicken, as seen in murine typhoid models during growth in liver and spleen (Janakiraman and Slauch, 2000; Boyer et al., 2002; Zaharik et al., 2004; Cunrath and Bumann, 2019). It is possible that increased proliferation of *Salmonella* seen in murine infections models may have higher iron demand which could not be satisfied by the high affinity iron acquisition systems. We did not observe a proliferation of any SEn strain during infection of chicken macrophage like cells, rather the wildtype cell numbers dropped by 2 logs at 24 h p.i. (Figure 6A). Increased proliferation in organs such as liver and spleen during systemic infections by NTS in chicken is not a characteristic feature compared to murine infections. So high affinity iron acquisition may be sufficient to maintain SEn in liver and spleen tissue as long as there is a lack of hyper-replicative phenotypes in chicken. However, Nagy et al. (2013), were able to show that ferric iron uptake utilizing catecholate siderophores is critical during persistent infection by STm (Nagy et al., 2013). In their study the authors have used a more natural route of infection (orogastric) and persistent infection was attained using mice which are genetically resistant to *Salmonella* (Nrmap1^+/+^) (Nagy et al., 2013). Authors confirmed that mutation in FepB caused a complete attenuation in which the Δ*fepB* strain was not recovered from any tissue examined after 2 weeks of infection (Nagy et al., 2013). FepB (ferrienterobactin binding protein) is the periplasmic binding protein for shuttling ferric iron-loaded siderophores into the bacterial cytoplasm (Figure 1A). In parallel, in mice challenged with a 1:1 ratio, the wildtype STm significantly outcompeted the Δ*fepB* strain regarding colonizing of liver, spleen, GALT and cecum at 2 weeks p.i. (Nagy et al., 2013). Importantly, their findings proved the fact that requirement of iron can be attributed to the duration of infection and the genetic background of the mice. Especially, during Nramp-1 mediated metal starvation, ferrous iron will be eventually limited, and intracellular *Salmonella* will depend on hijacking ferric iron sources from permissive niches. Also increased expression of major ferrous iron exporter protein ferroportin -1 has been evident during persistent infection models which in turn depleted iron pools in macrophages (Brown et al., 2015). In this regard Fe^3+^ uptake via catecholate siderophores appears to be pivotal for the survival of an intra-macrophage population of *Salmonella* during persistent infection. In contrast to mammals (humans and mice), persistent infection in extraintestinal sites is not part of NTS pathogenesis in chickens (Menanteau et al., 2018; Gal-Mor, 2019; Foster et al., 2021).

The cecum is the major predilection site for NTS serovars after fecal-oral infection in chickens. Digested ingesta retain up to 20 h in the cecum which provides a convenient habitat for SEn in chicken to persist and probably leading to the development of a carrier state (Menanteau et al., 2018). The anaerobic environment may favor ferrous over ferric iron on the apical surface of the cecal lumen for *Salmonella*. Indeed, the Δ*entB* strain was equally competitive as the wild type in colonizing cecum (Figure 2A). However, lack of FeoABC alone did not cause a fitness defect indicating that ferrous ion uptake via the FeoABC transporter was not essential during colonization of the cecum by SEn (Figure 3A). In contrast, the wildtype SEn was able to outcompete the Δ*feoΔentB* strain at all time points examined (Figure 2C), which again confirmed the nature of functional redundancy between two iron uptake pathways during cecal colonization. The margin of fitness defect was less than 10-fold at day 1 p.i. but increased over 10-fold toward day 7 p.i. (Figure 2C). Overall, the Δ*feoΔentB* strain showed a downward trend in persistence in the cecum milieu arguing to the role of low affinity iron system in fully rescuing the fitness defect as seen in some of the typhoid models. SEn can be attached to the mucosal epithelial cells, invade deeper tissues, and undergo transepithelial migration. The bacteria also extrude from epithelial cells to the lumen for excretion with feces. According to our experience, sampling cecal content for enumeration mainly quantify bacteria in the lumen, epithelia cells and to some extent lamina propria. To accurately determine bacterial localization to Payer’s patches, lamina propria and deeper tissues, immune-histochemistry staining of different segments in the cecum will be more valuable. Sampling the cecal content is superior to the enumeration of fecal pellets, because the latter does not represent the “true” cecal environment which is the major site of colonization and persistence in chickens. Redundancy of the high affinity iron uptake during gastrointestinal colonization was also observed by Tsolis et al. (1996). The authors were able to show that fecal shedding of a STm strain carrying mutations in both *feo* and *tonB* dependent ferric iron uptake (Δ*feoΔtonB)*, was significantly affected from day 1 p.i. of mice compared to single mutants (Δ*feo* or Δ*tonB*, respectively) challenged via intra gastric route mixed with wildtype STm (Tsolis et al., 1996). Taken together, bioavailability of iron in the gastrointestinal milieu can be extremely low and depends on high affinity iron acquisition to allow efficient colonization by *Salmonella* in both mice and chicken models. Findings from a recent attempt to analyze nutrient requirements for growth of intracellular STm during infection using HeLa epithelial cells suggested the presence of two populations of *Salmonella* with distinct demands for iron and manganese (Röder et al., 2021). In that study, the cytosolic fraction of STm showed hyper-replication compared to vacuolar *Salmonella* and this correlated with stronger induction of the *sitABCD* promoter during infection (Röder et al., 2021). It is highly suggestive that low affinity iron uptake systems may play an important role in certain populations of metabolically active *Salmonella* within the gut and even in the extraintestinal niches to fulfill the iron requirement. However, further experiments are needed to rule out expression of other high affinity iron uptake systems in those sub- populations of *Salmonella* to conclude whether they are dependent on low affinity iron uptake systems. It is plausible that in this hyper-replicative state, *Salmonella* may drive metabolism more toward manganese dependence as observed in *E. coli* (Sobota and Imlay, 2011). Previously, it has been documented that the upregulation of *sitABCD* transcripts (compared to broth cultures) of STm were among a unique set of virulence determinants during replication in the cecal mucosal wall of 1-day-old chicken at 16 h post infection (Harvey et al., 2011). Whether those observations pointed to hyperreplicative cytosolic fractions of *Salmonella* with unique metabolism during infection in 1-day-old chicken is unknown.

More detailed assessment of iron related factors involved in the virulence of *Salmonella* in birds is more complicated, especially in the cecal environment. In a single infection scenario, when present at extraintestinal sites such as liver or spleen, strains carrying deletions in siderophore synthesis (Δ*entB or ΔfeoΔentB)* cannot acquire any iron via uptake of enterobactin or salmochelin, because it does not synthesize any and no other “producers” are in the same location. This situation, however, changes when non-producers in the intestine or cecum are together with a microflora composed a number of species which can produce and secrete various type of siderophores. These include catecholate type (produced by avian pathogenic *E. coli*, *Proteus*, *Citrobacter* sp.) or ferrichrome (produced by various fungi). Those siderophores then can serve as an iron supply for the siderophore null strains. Similarly, in a co-infection scenario wildtype SEn strain can cross-feed iron-loaded siderophores to siderophore null strains (Δ*entB or ΔfeoΔentB)* rescuing fitness defect. However, such win scenarios for “cheaters” depend on a number of factors and assumptions, such as “cheaters” will be always be in close proximity to the wildtype, the wildtype will continuously produce siderophores, and there will be no sequestering of iron by other type of siderophores produced by the microflora (Kramer et al., 2020). Therefore, it is highly unlikely that those criteria will always be met in various chicken milieus. We observed that even when supplied with the maximum opportunity to be cross-fed by a wildtype strain, Δ*feoΔentB* did not gain fitness comparable to wild type in terms of expression of the luminescence signal (Supplementary Figure 5). In low-iron environments Δ*feoΔentB*_c10lux.CmR_ showed significantly low luciferase expression reaching to a maximum median value of ∼430 CPS value which is significantly low compared to WT_c10lux.CmR_ (∼17,000 CPS) (Supplementary Figure 5C). When cross-fed with a WT_CmR_ strain, Δ*feoΔentB*_c10lux.CmR_ showed only a small improvement, gaining a maximum of ∼1000 CPS. This amount of slight gain remained insignificant and low compared to the luciferase expression by WT_c10lux.CmR_ in a similar cross- feeding scenario (Supplementary Figure 5C). Though under nutrient-rich conditions (grown in LB medium), the defect of luciferase expression by Δ*feoΔentB*_c10lux.CmR_ was fully rescued by the cross-feeding of WT_CmR_ (Supplementary Figure 5C). The bioluminescence signal was dependent on bacterial cell densities and metabolic activity of the strain because it was driven by a constitutive promoter. Hence, our results strongly support that cross-feeding of non-siderophore producers by a wildtype strain will not always be able to rescue the fitness of the non-producer comparable to wildtype strain under certain conditions such as low-iron environments. Future co-infection experiments using mutants lacking specific receptors for siderophore uptake in addition to mutation to siderophore synthesis (ex: Δ*entBΔfepA, ΔentBΔfepAΔiroN, ΔentBΔfepAΔiroNΔcir*) will further help to eliminate these possible cross-feeding event *in vivo*.

Chicken’s innate immune response toward SEn infection might also play a role in the regulation of iron uptake systems. NTS strains such as the SEn do not induce a massive inflammation during colonization in the gastrointestinal tract or extraintestinal sites in chicken (Withanage et al., 2005; Setta et al., 2012). In line with that, we did not see inflammatory lesions in formalin-fixed tissues samples from duodenum, ileum, and cecum, except for few intraepithelial migrations of heterophils (data not shown). It is well known that inflammation-associated cellular responses can lower bioavailability of iron for pathogens. For example, a signaling cascade induced in the tissue by pro-inflammatory/inflammatory cytokines will lead to downregulation of iron absorption by enterocytes as seen in anemia of inflammation (Wessling-Resnick, 2010; Weiss et al., 2019). Secreted catecholate siderophores, on the other hand, can potentiate inflammation by stabilizing hypoxia inducible factor-1 alpha, which can be beneficial for certain pathogens for systemic spread (Holden et al., 2014, 2016). In fact, the importance of salmochelin has been subjected to the level of inflammation in the gut during STm infection in mice (Raffatellu et al., 2009). The avian immune system generally has lower responsiveness to LPS compared to mammals governed by polymorphisms in certain toll-like receptors (He et al., 2006; Higgs et al., 2006). We hypothesize that due to lack of prominent inflammation during SEn infection, chicken may have adapted to not rely solely on one mode of iron acquisition. This may lead to redundancy in utilizing high affinity iron acquisitions as observed by our experiments. Future experiments are warranted to delineate the possible link between siderophore production and to the level of inflammation seen in NTS infection in chicken.

*Salmonella* regulates iron homeostasis (and therewith the expression of iron uptake systems) by sensing a low intracellular iron environment using the Fur protein. The iron sensing molecular switch that has been introduced upstream of the *lux* operon contained the binding sequence for Fur which strongly binds under high iron conditions (Figure 7). Our data indicated that iron will be a limiting factor during colonization in chicken cecum as revealed by Fur-mediated de-repression of the *luxCDABE* reporter during *ex vivo* imaging of 1-day-old birds (Figure 8B). This iron sensing molecular switch contained a perfect Fur box sequence upstream of the *lux* genes which showed stronger binding affinity to the Fur protein in iron sufficient conditions and blocked the transcription of *lux* genes (repression). Hence, only mean value of 490 counts per second (CPS) of light intensity was generated by the Fur-box containing reporter, during growth in iron replete conditions (Figure 8A). This amount is 160 times lower than in a strain carrying constitutively expressed sig70c35 promoter. Even addition of 1mM of ferrous iron chelator dipyridyl (DP), to the LB medium did not fully de-repress the reporter to the level of signal intensity generated by the constitutively expressed reporter (80,000 CPS) *in vitro* (Figure 8A). However, the *lux* reporter expression of WT_c35Furbox lux.CmR_ positively correlated with the level of iron deprivation by adding DP to the growth medium (Figure 8A). For example, addition of 200 μM of DP increased the signal intensity to a mean value of 1,437 CPS value (3 X) and addition 400 μM DP raised the intensity to a mean value of 5,593 CPS (11 X) (Figure 8A). Hence, we used this information to estimate the magnitude of *in vivo* iron deprivation in terms of the amount of iron chelation by DP (Supplementary Data File 1). According to the estimated fold changes calculated by imaging data, at 48 h of infection, the mean fold change was slightly improved to a ∼2-fold value (1.9) compared to 24-h p.i. (1.1) (Figure 8C). One of the birds showed a sixfold change in Fur mediated de-repression at 48-h p.i. which was equivalent to iron deprivation created by addition of DP > 200 μM but less than 400 μM of DP (Figure 8C). Fur protein will be in bound and unbound status to the promoter depending on the cytosolic iron pool in each individual bacterium, giving rise to a mixed batch of birds with detectable signal from the cecum at each time point. Some populations of *Salmonella* will be able to resolve the amount of iron needed and may shut down the expression of the reporter. For example, at 48-h p.i. 3 out of 6 birds did not show regions of detectable bioluminescent signals in the cecum (Figure 8B). In contrast, the birds challenged with SEn carrying a constitutively expressed promoter, showed generally a homogeneous distribution of bioluminescent signals in ceca, from all the birds examined at 24- and 48-h of p.i. (Supplementary Figure 4A). This, in turn, shows that birds which did not show a bioluminescent signal might have had signals below the detection limit. The estimated upper margin of the detection limit was ∼677 CPS at *Salmonella* densities represented by 24- and 48-h p.i. This was estimated by the bird which showed the lowest detectable signal at 48-h p.i. from the cecum (bird number 121, see Supplementary Data File 1). In other words, a bioluminescent reporter generating less than ∼677 CPS of maximum signal intensity defined by *in vitro* plate reader (96 well, 150 μl) will not be able to give rise to a detectable bioluminescent signal at cell densities of ∼10^8^ CFU/g of SEn strains in the cecal compartment (subjected to exposture; 60 s, binning; medium in 1-day old birds). This estimated detection limit is also slightly higher than Fur mediated de-repression of the reporter grown under iron rich conditions (490 CPS). Hence, a detectable bioluminescent signal acquired by *ex vivo* imaging strongly indicated that SEn underwent a certain degree of iron starvation mediated by host’s nutritional immunity. *Salmonella* strains used for oral gavage were grown in iron replete conditions and most likely have a “reserve “of iron that can be mobilized from a cell internal iron pool, e.g., bound to bacterioferritin. So, there is a possibility that *Salmonella* will be able to resist host’s nutritional immunity status, to a certain generation time. Taken together, the bird that showed a bioluminescent signal as early as 2-h p.i. might have undergone a certain degree of iron starvation (Figure 8B). Future experiments can be extended to analyze *in vivo* promoter expression, belonging to high-affinity iron uptake systems utilizing bioluminescent reporters. However, *in vivo*, or *ex vivo* imaging of chicken poses an intrinsic challenge due to large size of the birds relative to mice which quenches most of the signal before reaching the surface. In the future, improved reporter systems with more infrared wavelength would facilitate the detection of signals from extraintestinal sites such as liver and spleen *in vivo*. This will allow us to provide more in-depth gene expression analysis in different localized niches as well as live whole-animal imaging.

In conclusion, here we report that high affinity iron acquisition (1) Fe^3+^ uptake profiting from catecholate siderophore synthesis (enterobactin and salmochelin) and (2) via Fe^2+^ uptake using the FeoABC transporter, provided a competitive advantage to SEn in cecal colonization, rapid systemic spread, and survival in extraintestinal sites in chickens. Both mechanisms showed functional redundancy and it seemed that iron acquisition using other (e.g., low affinity) uptake systems was not able to fully rescue the fitness defect at all time points examined. Fur mediated de-repression of a bioluminescent reporter examined by *ex vivo* imaging of the gastrointestinal tract revealed that even at early colonization events (2 h p.i.) *Salmonella* already undergoes a considerable iron starvation in the cecal compartment which, in turn, can shape some of the iron regulation mechanisms in the chicken. We believe that the unique avian immune systems may contribute to the redundant nature of the high-affinity iron acquisition systems of SEn infections in chicken. Hence, it will be pivotal to understand the regulation of *Salmonella* iron homeostasis in relation to the avian immune system in order to formulate therapeutics targeted at poultry farming operations to minimize contamination.

## Materials and Methods

### Bacterial Strains, Media, and Growth Conditions

*Salmonella* Enteritidis used in this study was isolated from poultry during an outbreak in Canada. It was provided by the Canadian Food Inspection Agency. The strain was labeled as LS101 and maintained in a glycerol stock at VIDO-University of Saskatchewan. Virulence potential of this strain is currently unknown and whole genome sequencing assembly is yet to be conducted. Cloning experiments were performed in *Escherichia coli* DH10B or *Escherichia coli* CC118 (Herrero et al., 1990). *Escherichia coli* DH5α used as a negative control to infect in HD11 cells.

Plasmid purification, was done using Qiagen MiniPrep kit and strains used for cloning were inoculated from frozen stocks into Luria agar supplemented with the appropriate antibiotics (chloramphenicol; Cm 10 μg mL^–1^, carbenicillin; Cb 50 μg mL^–1^, or both) and grown overnight at 37°C or 30°C. Vectors; pCS26-Cm, pHSG415, and pUC18R6K-mini-Tn7T utilized in this study were kindly provided by Dr. Aaron White, VIDO, University of Saskatchewan (Shivak et al., 2016). Single colonies were selected from each plate to inoculate 5–10 ml mL LB broth cultures and proceeded with plasmid purification steps according to manufacturer’s guide. Quality and purity of the DNA was checked by “Nanodrop.”

Bioluminescence assays were conducted as described previously using either Victor X^3^ or Victor V^3^ multilabel plate reader (Perkin-Elmer) (Shivak et al., 2016). Briefly, overnight cultures of SEn strains carrying the *lux* reporter, were diluted 1:600 in LB broth supplemented with or without antibiotics (10 μg ml^–1^ Cm) to a final volume of 150 μL in 96-well clear-bottom black plates (9520 Costar; Corning Inc., fisher scientific, Canada). The culture in each well was overlaid with 50 μL of mineral oil prior to luciferase assays to avoid evaporation of the growth media. The plate reader measured absorbance (598 nm, 0.1 s), and luminescence (1s exposure) every 30 min during growth at 37°C with agitation.

2,2′-Bipyridyl (CAS 366-18-7, Sigma-Aldrich, Milipore Sigma, Oakville, ON, Canada) was used to chelate iron in the medium. 1.36 mg of 2,2’-Bipyridyl was completely dissolved in 100 ml of double distilled water by gently warming to gain a final concentration of 20 mM (stock solution). Then it was subjected to sterile filtration. This stock solution was used for generating subsequent concentrations of 2,2′-Bipyridyl solutions used in experiments.

*Salmonella* Enteritidis challenge inoculums were prepared from a frozen stock propagated on LB plates with appropriate antibiotic (Cm 34 μg mL^–1^). Single colonies were grown overnight in 5–10 ml of LB medium with Cm at 37°C with agitation. Overnight cultures were then subcultured (1:100) in LB at 37°C with agitation to gain OD 0.7 in the exponential growth phase. The Δ*feoΔentB* strain was slow to grow in LB media hence overnight cultures were grown to 36-h period before subculture (1:50).

### Chromosomal Transposition of the Reporter -*luxCDABE* Generation of Iron Responsive Reporter Construction

The *lux* operon *CDABE* from *Photorhabdus luminescens* was used as the reporter for integration into the chromosome of the SEn LS101 strain. The method is based on the minitn7T transposon system developed by Shivak et al. (2016). Briefly, pHSG415 (12 kb), temperature sensitive plasmid (helper vector) encoding transposon genes (*tns-ABCD*), was electroporated (800Ω, 200 μV, 2 mm cuvette) into SE strains. After electroporation cells were recovered in SOC medium (2% tryptone, 0.5% yeast extract, 10 mM NaCl, 2.5 mM KCl, 20 mM Mg, 20 mM glucose) for 2 h with agitation (200 rpm) at 30°C. Colonies were grown from LB plates supplemented with Cb at 30°C. Competent cells harboring pHSG415-*tnsABCD* were prepared by sedimentation (7,000 rpm, 10 min), washed three times by 10% glycerol. Then these competent cells were used for another electroporation with 100–200 ng of pUC18R6K-mini-Tn7T (delivery vector) plasmid. The delivery vector harbors the *lux* operon with suitable promoters (σ^70^ c35 or c10 in reverse orientation). Following recovery in SOC medium, bacterial cells were agitated at 30°C for another 2 h. During this period, the *lux* operon will be removed from the delivery vector and integrated at high frequency into the *attTn7* site of bacteria by transposition. Transformed cells were recovered on Cm LB agar plates at 37°C overnight promoting the removal of the temperature sensitive pHSG415. Chromosomal integration of the reporter fragment into the *attTn7* site downstream of the *glmS* gene of SEn was confirmed by PCR using primers described previously (Shivak et al., 2016). Primers have been designed to target the *glmS* gene (glmsFw, glmsRw), lux genes (Lux check), Cm marker (Cm-check) are shown in Supplementary Table 2.

### Cloning Method to Generate Molecular Switch Responsive to Iron

The backbone of the promoter was chosen as the sigma factor 70 recognized sequence. RNA polymerase is directed to either –35 region or –10 region of these type of promoters for transcription. Forward and reverse phosphorylated oligonucleotides containing a perfect “Fur box” sequence at –10 region **(GATAATGATAATCATTATC)** were mixed (2 μl, 100 μM) with ligation buffer (5 μl with ATP) in 50 μl reaction, heated to 80°C for 7 min and allowed to cool slowly to room temperature. These oligos were designed to contain *Xho*I/*Bam*HI overhangs (Supplementary Table 2). In the mixture containing annealed products, directly used in a ligation reaction with *Xho*I/*Bam*HI-digested pCS26-Cm vector as described in reference (Shivak et al., 2016). Colonies with successful ligation emitted a bioluminescent signal which was detected by IVIS Lumina II *in vivo* imaging system (PerkinElmer, auto exposure settings). Purified pCS26-Cm with promoters were sequenced by PZE05 and PZE06 primers for correct insertion upstream of *luxCDABE operon* (Sanger sequencing by Plant Biotechnology Institute, University of Saskatchewan). pCS26 reporter plasmids were digested with *Pac*I and ligated into *Pac*I-digested pUC16R6K mini-Tn7T vector carried in CC118 cells. Potential clones of *E. coli* CC118 harboring the mini-Tn7T were selected by growth on LB agar supplemented with 50 μg ml^–1^ Cb and 10 μg ml^–1^ Cm and screened for bioluminescent signal as mentioned earlier. This resulted in two orientations of the reporter, but we utilized reverse orientation. In the reverse orientation, *luxCDABE* is directed from Tn7 left to Tn7 right in the plasmid (Shivak et al., 2016). To confirm orientation, purified plasmid was digested by *Not*I -HF, *Xba*I, *Eco*RV-HF enzymes and the band pattern was visualized in a 1% agarose gel (4, 3.8, 1.6, 1.2 kb, 329 bp) after electrophoresis. Finally, the delivery vector was used for chromosomal integration of the reporter into SEn as described above. Luciferase assay was performed (as described earlier) for selected clones, under iron-rich and depleted medium to check whether expression of *lux* operon was repressed or de-repressed by Fur under those conditions, respectively.

### Generation of Mutants Containing Antibiotic Marker

The Lambda Red system which was described by Datsenko and Wanner (2000) was utilized to create gene knockouts and replacement by an antibiotic marker (Cm). Briefly, strain LS101 was transformed with temperature sensitive pKD46 which encoded red recombinase enzymes Δ*,β* and *exo* induced under L-arabinose. Fresh, electro-competent cells containing pKD46 (50 μl of aliquots, induced by L-arabinose) were made to be used for subsequent transformation of PCR products for gene replacement. Primers were designed based on the complete genome sequence of a PT4 *Salmonella enterica* subsp. *enterica* serovar Enteritidis strain deposited in NCBI (NC_011294) (Thomson et al., 2008). The PCR products generated gene replacement had the Cm marker from the pKD3 flanked by Flippases recognition sequence and 49-nucleotide homology extension of the gene of interest. Gel purified PCR products (100–500 ng) were electroporated in to LS101.pKD46 cells and cells recovered in SOC medium at 37°C for 2–3 h and plated in 9 μgmL^–1^ of Cm. Cells of colonies containing the anticipated mutations were subjected to PCR and PCR products were sequenced for further verification. The same strategy was used to create a LS101 isolate containing a Cm marker (Supplementary Table 2). The Cm marker was inserted downstream of the *glmS* gene which contains the *attTn7* site and has been utilized previously to obtain SEn with various antibiotic resistance (Desin et al., 2009).

### Chicken Infection Model Using *Salmonella* Enteritidis

Co-infection experiments were carried out in 1-week-old commercial broilers. One-day-old birds were obtained from local hatcheries and raised at the VIDO-animal care facility in direct accordance with guidelines drafted by the University of Saskatchewan’s Animal Care Committee and the Canadian Council on the Use of Laboratory Animals. Birds were screened for *Salmonella* on arrival by streaking fecal swabs onto brilliant green agar plates. A second screening was performed 2 days before oral challenge to check whether the chicks were free from *Salmonella*. Each bird was orally gavaged (needle size 18-gauge, 1.5 inch) with an inoculum containing 10^9^ CFU from each SEn strain (1:1 ratio) suspended in 0.5 ml of commercial phosphate buffered saline (1X, pH = 7.2, Gibco, ThermoFisher scientific, Canada). After challenge, birds of each co-infected group were raised in separate rooms as free run. No bedding material was changed during the experiment. Thirteen to fourteen birds were euthanized at days 1, 4, and 7 post challenge. Samples from the liver (part of the liver), spleen (whole organ), and cecal contents were weighed and homogenized in saline (0.85% sodium chloride), and serial dilutions were plated onto brilliant green agar plates (BGA-17 μg mL^–1^) to determine bacterial counts. In addition, aliquots from homogenates from liver and spleen were enriched by incubation in selenite broth overnight at 37°C. Enriched samples were streaked onto BGA to single colonies to determine whether they contained each *Salmonella* strain inoculated. For analysis of Fur-mediated depression of *lux* operon, SPF eggs were ordered form Charles River Laboratories and incubated for 21 days until hatch at VIDO. Once hatched they were acclimatized to the conditions for a 24-h period before challenge. Birds were challenged with an oral dose of 10^9^ CFU per bird and 5–6 birds from each time point were euthanized for *ex vivo* imaging by IVIS Lumina II animal imager (Caliper).

### *Ex vivo* Imaging by Whole Animal Imager

The gastrointestinal tract of each bird to be analyzed was laid out in a large petri dish to visualize the signal emitted from the surface of the organs. After initializing the instrument (IVIS Lumina II by Caliper Life sciences, PerkinElmer, United States), the bioluminescent setting was selected (Filter-open). To maximize capturing low bioluminescent signals, the aperture size was set to 1 (*f* = 1). All the signals were taken at the exposure of 60 s with medium binning to create the images. The radiance of each cecal compartment measured using the region of interest (ROI) tool provided by the Living imaging software (version 4.0). The ROI of the cecum was selected by drawing exact boundaries around the cecum using the free drawing tool and the radiance value was automatically generated by the software.

### Gentamicin Protection Assay Using HD11 Cells

Chicken HD11 cells are macrophage like bone marrow derived cells transformed with the avian leukemia virus, MC29 (Beug et al., 1979). Chicken HD-11 cells were grown and maintained as previously described with some modifications (Wisner et al., 2011). Cells were maintained at 39°C, in a humidified incubator (5% CO2), in RPMI 1640 (with L-glutamine -Gibco) supplemented with 10% heat-inactivated, fetal-bovine serum (FBS). HD-11 cells grew vigorously after 1 week of taking them out from the stock. The growth medium was changed and cells passaged accordingly (without trypsinization) every 2–3 days when they got 70–80% confluent using a new 75 cm^2^ flask. HD-11 cells are generally circular in shape and more like suspension cells. The assay was conducted using a 24-well Cell-Bind plate (Corning, fisher scientific, Canada) in order to obtain a good adherence to the surface. Each well was seeded with 5 × 10^5^ cells and stimulated with 10 ng μL^–1^ of phorbol 12-myristate 13-acetate (PMA) (Sigma-Aldrich, Milipore Sigma, Oakville, ON, Canada) to differentiate to macrophage like appearance (16 h). Differentiation conditions of monocytes to macrophages using PMA have been studied using mammalian cells (Pinto et al., 2021), however a detailed analysis of similar maturation conditions of HD-11 cells is lacking. According to our experience, 10–20 ng μL^–1^ of PMA was sufficient to cause a morphological change (spindle formation/macrophage like appearance). Medium was changed to fresh RPMI 1640 (L-glutamine -Gibco and 10% heat inactivated FBS) without PMA after overnight of incubation period to rest for another 3 h before infection.

Bacteria grown in LB medium (OD = 0.7) were pelleted and suspended in pre-warmed RPMI 1640 (L-glutamine -Gibco). Then each well was infected at a multiplicity of infection (MOI) of 1 and incubated for 1 h at 39°C, in a humidified incubator (5% CO2). After 1 h, media containing bacteria were carefully removed from the HD-11 cells, and the cells were washed once with PBS (1X) containing 400 μg mL^–1^ of gentamicin. RPMI 1640 (L-glutamine -Gibco and 10% heat inactivated FBS) containing gentamicin (400 μgmL^–1^) was then added to each well, and the plates were placed back at 39°C. At each time point (3′ and 24-h post infection), medium was removed, and cells were washed once with PBS (without antibiotics), and lysed in 0.5 ml 1% Triton X-100 in PBS. A dilution series of the cell monolayer fractions were made and 100 μl of each dilution (10^–3^, 10^–2^, 10^–1^, 0) was plated onto LB agar plates using borosilicate glass beads. Plates incubated overnight at 37°C. Strains carrying a mutation in enterobactin synthesis genes were incubated an additional time of 16 h at room temperature.

### Nitric Oxide Measurements

Nitric oxide generated in HD-11 cells during *Salmonella* infection was measured by Griess assay kit according to the guidelines provided by the manufacturer (Thermo Fisher-G-7921). The reaction measures more stable metabolite of NO, nitrite (NO_2_^–^) (Griess, 1879). 150 μl of the media fraction at each time point (3 and 24 h) were removed and incubated (30 min) with the master mix containing Griess reagents (20 μl Griess reagent + 130 μl of deionized water) in a 96 well plate (Costar). Absorbance was measured at 548nm of wavelength using a plate reader at the end of the incubation period. In each assay, standard curve generated measuring absorbance of sodium nitrite standards (100, 50, 25, 12.5, 6.25, 3.125, 1.5 μM).

### Data Analysis

All the statistical data analysis and graphs created using GraphPad Prism software version 8.4.2 under institutional license were provided to the Vaccine and Infectious Disease Organization. Non-parametric tests were used in selecting the appropriate test method because of data is not normally distributed. The competitive index score was calculated by {(Mutant/Wildtype) CFU/g_*output*_} ÷ {(Mutant/Wildtype) CFU_*inoculum*_}. CI values were analyzed using Wilcoxon Signed Rank test which compared the median difference of the sample to theoretical value of 1. Mean comparison of the colony counts between the wild type and mutant was performed using Kruskal–Wallis test. Mann–Whitney was used to compare the median difference of the colony counts when mentioned. Fisher’s exact test was used to analyze enrichment data using fractions of birds infected and non-infected. The statistical significance of all the methods was set at *p* = 0.05.

## Data Availability Statement

The datasets presented in this study can be found in online repositories. The names of the repository/repositories and accession number(s) can be found in the article/Supplementary Material.

## Ethics Statement

The animal study was reviewed and approved by University of Saskatchewan’s Animal Care Committee and the Canadian Council on the Use of Laboratory Animals.

## Author Contributions

DW and WK designed the study and wrote the manuscript. DW, P-KL, and WK collected the experimental data. DW, P-KL, WK, and SG analyzed the data. BA and AW discussed the data and edited the manuscript, DW created the figures. All authors contributed to the article and approved the submitted version.

## Conflict of Interest

The authors declare that the research was conducted in the absence of any commercial or financial relationships that could be construed as a potential conflict of interest.

## Publisher’s Note

All claims expressed in this article are solely those of the authors and do not necessarily represent those of their affiliated organizations, or those of the publisher, the editors and the reviewers. Any product that may be evaluated in this article, or claim that may be made by its manufacturer, is not guaranteed or endorsed by the publisher.

## Supplementary Material

Supplementary Figure 1 Percentage of birds positive for spleen and liver infection after enrichments. Dark column represents % birds for infected by the strain which are recovered by liver/spleen enrichments. Non-infected % of birds presented by shaded gray. Fisher’s exact test used for statical analysis. ns, not significant.

Supplementary Figure 2 Infectivity of bioluminescent strains vs. non-bioluminescent strains as obtained by liver and spleen enrichments. Dark column represents% birds for infected by the strain which are recovered by liver/spleen enrichments. Non-infected % of birds presented by shaded gray. Fisher’s exact test used for statical analysis. ns, not significant.

Supplementary Figure 3 Percentages of live and dead HD-11 cells at 24-h post infection. Triplicate of cells pooled and harvested from Cell Bind*^R^* plates after 24-h infections. 0.4% Trypan blue solution was used to differentiate dead and live cell by visualization under the microscope. Cells were counted by hemocytometer. This result is from one assay from activated HD-11 passage number 6.

Supplementary Figure 4 **(A)** *Ex vivo* imaging of the gastrointestinal tract. One-day-old SPF were orally gavage with WT_*c35 lux.CmR*_ strain. Signal intensity in counts shown in rainbow color scale. Minimum value is represented by purple color range while maximum represented by toward red color. **(B)** Bacterial load in the cecal content after infection from the two reporter strains of SEn. Colony counts indicated in log_10_ scale. Each symbol represents a bird at a time point. Median value of the data set is shown by horizontal bar. Mean difference was accounted for statistical analysis. ns, not significant.

Supplementary Figure 5 Kinetic of *lux* operon expression of a siderophore null strain (Δ*feoΔentB*) during a cross-feeding assay. **(A)** Iron deplete conditions: heat inactivated bovine calf serum with 1 mM of dipyridyl. **(B)** Iron replete conditions: LB medium. Luminescence measured by Victor V3 plate reader. Expression is in log scale of counts per second (CPS).) Starting culture were grown in LB and adjusted to OD600 = 1. Mixed: wildtype to Δ*feoΔentB* (ΔΔ) ratio of 1. Assay was performed in triplicates for four times. Each line represents a technical replicate. Arrow indicates the cross-feeding event. **(C)** Maximum luminescence value (Lumi_*max*_) by each reporter. Maximum CPS values obtained from **(B)**. Each symbol represents a technical replicate. Mean differences were analyzed using appropriate test method. ns, not significance. Significance set at 0.05. ^****^*p* ≤ 0.0001, ^**^*p* = 0.0057.
