# Supplementary material for: High Affinity Iron Acquisition Systems Facilitate but Are Not Essential for Colonization of Chickens by Salmonella Enteritidis
Source: Front Microbiol. 2022 Mar 3;13:824052. doi: 10.3389/fmicb.2022.824052 (PMC8928163; doi:10.3389/fmicb.2022.824052)
Supplement: Supplementary Figure 1 — Percentage of birds positive for spleen and liver infection after enrichments. Dark column represents % birds for infected by the strain which are recovered by liver/spleen enrichments. Non-infected % of birds presented by shaded gray. Fisher’s exact test used for statical analysis. ns, not significant. [file Data_Sheet_1.docx]

**Appendix:** supplementary figures and tables

**Figure 1: Percentage of birds positive for spleen and liver infection after enrichments.** Dark column represents % birds for infected by the strain which are recovered by liver/spleen enrichments. Non-infected % of birds presented by shaded grey. Fisher’s exact test used for statical analysis. ns: not significant.

**Figure 2: Infectivity of bioluminescent strains vs non-bioluminescent strains as obtained by liver and spleen enrichments**. Dark column represents % birds for infected by the strain which are recovered by liver/spleen enrichments. Non-infected % of birds presented by shaded grey. Fisher’s exact test used for statical analysis. ns: not significant.

**Figure 3: Percentages of live and dead HD-11 cells at 24-hour post infection.** Triplicate of cells pooled and harvested from Cell Bind^R^ plates after 24-hour infections. 0.4% Trypan blue solution was used to differentiate dead and live cell by visualization under the microscope. Cells were counted by hemocytometer. This result is from one assay from activated HD-11 passage number 6.

**A**


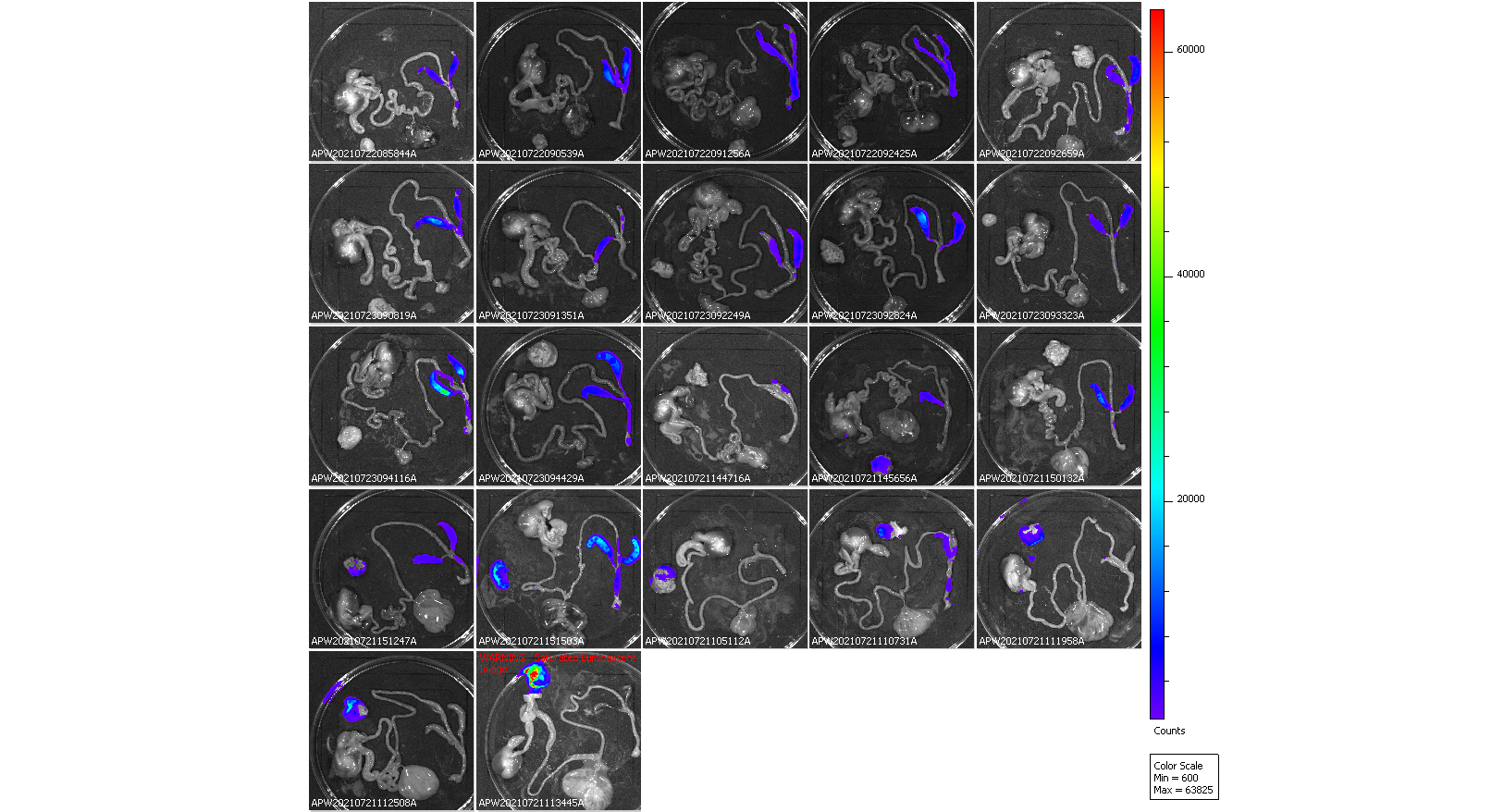


**48 hr p.i.**

**48 hr p.i.**

**48 hr p.i.**

**48 hr p.i.**

**48 hr p.i.**

**48 hr p.i.**

**48 hr p.i.**

**24hr p.i.**

**24hr p.i.**

**24hr p.i.**

**24hr p.i.**

**24hr p.i.**

**24hr p.i.**

**6 hr p.i.**

**6 hr p.i.**

**6 hr p.i.**

**6 hr p.i.**

**6 hr p.i.**

**2 hr p.i.**

**2 hr p.i.**

**2 hr p.i.**

**2 hr p.i.**

**B**

**Figure 4: A) Ex-vivo imaging of the gastrointestinal tract.** 1 day-old SPF were orally gavage with WT_c35 lux.CmR_ strain**.** Signal intensity in counts shown in rainbow color scale. Minimum value is represented by purple color range while maximum represented by towards red color. **B) Bacterial load in the cecal content after infection from the two reporter strains of SEn.** Colony counts indicated in log_10_ scale. Each symbol represents a bird at a time point. Median value of the data set is shown by horizontal bar. Mean difference was accounted for statistical analysis. ns: not significant.

.

**Figure 5: Kinetic of *lux* operon expression of a siderophore null strain (*∆feo∆entB*) during a cross-feeding assay** . **A) Iron deplete conditions**: heat inactivated bovine calf serum with 1mM of dipyridyl. **B) Iron replete conditions:** LB medium. Luminescence measured by Victor V3 plate reader. expression is in log10 scale of counts per second (CPS).) Starting culture were grown in LB and adjusted to OD600 =1. Mixed: wildtype to *∆feo∆entB* (∆∆) ratio of 1. Assay was performed in triplicates for four times. Each line represents a technical replicate. Arrow indicates the cross-feeding event. **B) Maximum luminescence value (Lumi_max_) by each reporter.** Maximum CPS values obtained from figure B. Each symbol represents a technical replicate. Mean differences were analyzed using appropriate test method. ns; not significance. Significance set at 0.05. ****p=<0.0001,** p=0.0057.

**A**

**B**

**C**

**Table 1: Bacterial strains used in this study**

| Bacterial strain | Description and/or genotype | Reference |
| --- | --- | --- |
| LS101(WT) | Salmonella enterica serovar Enteritidis wild type | Unpublished data |
| LS190 (WT_c35 lux.CmR_) | LS101, *att* Tn7::sig70c35 *luxCDABE.cat* | This study |
| LS278 (WT_c10 lux.CmR_) | LS101, *att* Tn7::sig70c10 *luxCDABE.cat* | This study |
| LS289 (WT_CmR_) | LS101, *att* Tn7:: *cat* | This study |
| LS229 (*∆feo _CmR_*) | LS101*, ∆feoABC*::*cat* | This study |
| LS230 (*∆feo*) | LS101, *∆feoABC*::no marker | This study |
| LS236 (*∆feo_c35 lux.CmR_*) | LS101, *∆feoABC att Tn7::* sig70c35 *luxCDABE.cat* | This study |
| LS231 (*∆entB _CmR_*) | LS101, *∆entB*::*cat* | This study |
| LS234 (*∆entB*) | LS101, *∆entB::*no marker | This study |
| LS290 (*∆entB* _c10 lux.CmR_) | LS101, *∆entB att* Tn7*::* sig70c35 *luxCDABE.cat* | This study |
| LS235 (*∆iroB*) | LS101, *∆iroB*::no marker | This study |
| LS246 (*∆feo∆iroB _CmR_*) | LS101, *∆feoABC∆iroB::cat* | This study |
| LS249(*∆feo∆iroB* ) | LS101, *∆feoABC∆iroB::*no marker | This study |
| LS284 (*∆feo∆entB _CmR_* ) | LS101*, ∆feoABC∆entB::cat* | This study |
| LS280 (*∆feo∆entB)* | LS101*, ∆feoABC∆entB::*no marker | This study |
| LS295 (*∆feo∆entB _c10 lux.CmR_)* | LS101*, ∆feoABC∆entB att Tn7::* sig70c10 *luxCDABE.cat* | This study |
| LS204 (*∆tonB)* | LS101*, ∆tonB::*no marker | This study |
| LS245 (WT _c35 Furbox lux.CmR_) | LS101, *att* Tn7::sig70c35 Fur box *luxCDABE.cat* | This study |
| *E. coli* DH5α | DH5α F− φ80dlacZΔM15 Δ(*lacZYA-argF*) *U169 deoR recA1 endA1 hsdR17*(rK− mK−) *phoA supE44 λ−thi-1 gyrA96 relA1* | Invitrogen |
| *E. coli* DH10B | F^–^*mcr*A Δ(*mrr*-*hsd*RMS-*mcr*BC) φ80*lac*ZΔM15 Δ*lac*X74 *rec*A1 *end*A1 *ara*D139 Δ(*ara-leu*)7697 *gal*U *gal*K λ^–^*rps*L(Str^R^) *nup*G | Invitrogen |
| *E. coli* CC118 | λ*pir* | 74 |

**Table 2: Primers used in this study**

| **Primer name** | **Sequence (5'-3')** | **Purpose** |
| --- | --- | --- |
| sig70-c35 Fur box FW | TCGAGAATAATTCTTGACATTTATGCTTCCGGCTCGGATAATGATAATCATTATCACTAAAAG | sig70-35c-fur promoter construct |
| sig70-c35 Fur box RW | GATCCTTTTAGTGATAATGATTATCATTATCCGAGCCGGAAGCATAAATGTCAAGAATTATTC |  |
| pZE05-RW (ref 23) | CCAGCTGGCAATTCCGA | To verify promoter sequence in pCS26 plasmid. |
| pZE06- FW(ref 23) | AATCATCACTTTCGGGAA |  |
| Lux-check (ref 23) | TCAACACTTGTTTCTTTGAGG | To verify chromosomal insertion of *luxCDABE* |
| glmS detect-FW (ref 23) | AACCACCCGTTCAGGCTGGCTA | To verify chromosomal insertion at *glm*S site |
| glmS detect-RW (ref 23) | ACGTTGACCAGCCGCGTAAC |  |
| Cm-check (ref 23) | CCCCGTGGAGGTAATAATTG | To verify chromosomal insertion of Cm^R^ marker of the reporter |
| feo lamda -FW | ATGCAATTCACTCCTGACACTGCGTGGAAAATCACCGGCTTTGCGCGTGA GTGTAGGCTGGAGCTGCTTC | To amplify PCR fragment containing chloramphenicol marker with FRT from pKD3 , flanked by extension of *feoABC* gene. |
| feo lamda -RW | TAACGCGGGATAAAGGCCGGATAAGACGCGTTACGTCGCTACCCGGCGAA ATATGAATATCCTCCTTAG |  |
| FeoABC-check FW | ATGCAATTCACTCCTGACAC | To sequence *feoABC* gene (2.8kb) and verify gene replacement by lambda recombinases |
| feoABC-check RW | ATAAGACGCGTTACGTCG |  |
| entB Lamda-FW | ATGGCAATCCCGAAACTACAGTCTTACGCGCTGCCCACCGCACTGGATAT GTGTAGGCTGGAGCTGCTTC | To amplify PCR fragment containing chloramphenicol marker with FRT from pKD3 , flanked by extension of *entB* gene. |
| entB Lamda-RW | TACTCTACCCCGCGAGAAAGCAGCGCCCACCAGGCGTCAATGGTCGGGT ATATGAATATCCTCCTTAG |  |
| entB-check FW | ATGGCAATCCCGAAACTACA | To sequence *entB* gene (858 bp) and verify gene replacement by lambda recombinases |
| entB-check RW | TTACTCTACCCCGCGAGAAA |  |
| iroB lamda-FW | TTCTCATTAATGACTTGTTCGATTTATGACGTGGAGAGAGAGGATTTCTC GTGTAGGCTGGAGCTGCTTC | To amplify PCR fragment containing chloramphenicol marker with FRT from pKD3 , flanked by extension of *iroB* gene. |
| iroB Lamda-RW | TGGCGACACACGCTGGGTTCAGCCGCCATATTGTCATTGCGCTGCCGCGG ATATGAATATCCTCCTTAG |  |
| tonB lambda-FW | ATGATTGCTATTTGCATTTAAAATTCAGCTCTGGTTTTT CAACTGAAACGCATATGAATATCCACCTTAG | To amplify PCR fragment containing chloramphenicol marker with FRT from pKD3 , flanked by extension of *tonB.* |
| tonB lamda-RW | CTTACGCCGCCAGCAGGTGATGGTATATTCCTACTG GCGGCGCCAGAGATGTGTAGGCTGGAGCTGCTTC |  |
